# Overlooked and undertreated: Hearing loss among individuals with intellectual disabilities – A German cohort study

**DOI:** 10.1371/journal.pgph.0006872

**Published:** 2026-08-19

**Authors:** Katrin Neumann, Anna Sophia Schwalen, Corinna Gietmann, Vincent Jankovic, Susanne Wasmuth, Martin Scharpenberg, Susanna Zielonkowski, Lukas Prein, Awa Naghipour, Anja Neumann, Katharina Schwarze, Werner Brannath, Karolin Schäfer, Jasmin Filip, Christian Speckemeier, Sarah Schlierenkamp, Sandra Werner, Silke Neusser, Anna Wiegand, Peter Matulat, Ruth Lang-Roth, Nicole Stuhrmann, Emmanouela Dimitrakopoulou, Nils Vogt, Ross Parfitt, Heike Weiler-David, Philipp Mathmann

**Affiliations:** 1 Department of Phoniatrics and Pediatric Audiology, University Hospital Münster, Münster, Germany; 2 Universitas Airlangga, Surabaya, Indonesia; 3 Competence Center for Clinical Trials Bremen, University of Bremen, Bremen, Germany; 4 Institute for Health Care Management and Research, University of Duisburg-Essen, Essen, Germany; 5 Institute for Special Needs Education (d/Deaf and Hard of Hearing), University of Duisburg-Essen, Germany; 6 Essener Forschungsinstitut für Medizinmanagement (EsFoMed) GmbH, Essen, Germany; 7 Department of Otorhinolaryngology, Head and Neck Surgery, Medical Faculty, University Hospital Cologne, Cologne, Germany; 8 Practice for Otolaryngology, Phoniatrics & Pediatric Audiology, Duesseldorf-Meerbusch, Germany; 9 Phoniatrics and Pediatric Audiology, Head and Neck Surgery, Helios HSK, Wiesbaden, Germany; 10 Division of Phoniatrics and Pediatric Audiology, Department of Otorhinolaryngology, Head and Neck Surgery, St. Elisabeth-Hospital, Ruhr University Bochum, Bochum, Germany; 11 Kampmann Hörsysteme GmbH, Bochum, Germany; 12 Department of Health Management, AOK Rheinland/Hamburg, Duesseldorf, Germany; 13 Department of Otorhinolaryngology, Head and Neck Surgery, Johannes Wesling Klinikum Minden, Ruhr-University Bochum, Minden, Germany; 14 South African College of Music, University of Cape Town, Cape Town, South Africa; PLOS: Public Library of Science, UNITED STATES OF AMERICA

## Abstract

People with intellectual disabilities (ID) are 5–10 times more likely to have hearing loss than the general population, yet it often remains undetected and untreated. To address this gap, the study *HörGeist* examined the feasibility, outcomes, barriers, and costs of an outreach program with hearing screening, diagnostics, intervention, and monitoring in everyday environments to implement a universal program in Germany. This report outlines ear and hearing care status before and after inclusion in the program and compares outcomes with standard care and a clinic-based invitation model. In a population-based, age-stratified cohort study, 1053 participants with ID (37% female; age 1–90 years) received screening and reference measurements in nurseries, schools, workplaces, and residences. When screening indicated possible hearing loss, on-site diagnostics and therapy were provided or referrals issued. A comparison cohort of 141 individuals with ID was invited to a clinic for identical procedures; none participated. All procedures were repeated after 1 year. Hearing loss was known in 14% of outreach participants at study entry; 8.7% had previously received hearing aids, but fewer than half (4.3%) used them. The program diagnosed hearing loss in 44% of participants, with 70% previously undiagnosed. After screening, 27% received therapy recommendations, but only 37% adhered to them (30% for hearing-aid prescriptions). Undertreated hearing loss declined only slightly, from 43% to 38%, over time. Major barriers included refusal by caregivers (31%) and participants (33%), often appearing to be linked to limited awareness of the treatability and consequences of hearing loss, as well as time and staff shortages. Outreach hearing screening programs are highly necessary, feasible, and effective in detecting hearing loss in people with ID, but substantial care gaps persist. Implementation requires public and professional awareness and rehabilitation strategies that include hearing and communication training and actively involve individuals with ID and their caregivers.

## Introduction

Approximately 1–2% of the global population are individuals with intellectual disability (ID) [1]. A meta-analysis by Maulik et al. (2011) reported the global prevalence as 10.37 per 1,000 individuals [2]. Rates are highest in low- and middle-income countries and are higher in males than in females as well as in children and adolescents than in adults [1,2]. Prevalence estimates for Germany fall within this internationally observed range [3,4].

Individuals with ID are less healthy than the general population. They experience higher rates of a wide range of diseases, such as vision and hearing loss, cardiovascular, dental, and musculoskeletal conditions, epilepsy, movement disorders, and obesity [4–15]. These comorbidities often begin earlier [6] and are accompanied by elevated mortality rates [5,12,16–20] compared to the general population. Population-based studies from the UK [21] and Denmark [20] have shown that individuals with ID are more than twice as likely to die from causes that could have been prevented or treated through appropriate healthcare than the general population. People with ID face a significantly higher risk of undetected health conditions. For instance, Baxter et al. (2006) [22] found that over half of individuals with ID who received health checks had previously-unidentified medical needs. In light of such evidence, the World Health Organization (WHO) has stated that individuals with ID are often disadvantaged in their health care and that improving healthcare for in particular children and young people with ID is a key priority in public health policy [23].

Individuals with ID also face a significantly increased risk of hearing loss, which occurs approximately five to ten times more frequently in this population than in the age-matched general population [24–29]. Hearing screening conducted during national and international Special Olympics events—the world’s largest sports training and competition for individuals with ID—have reported referral rates ranging from 22% to 38% [28,30–33]. These data provide a realistic picture of the hearing status of adolescents and adults with ID; a comparison between screening results and full audiological diagnostics showed a specificity of 98% and a sensitivity of 100% [28]. Similar rates of hearing loss have also been reported in children and adolescents with ID attending special education schools in Germany [27], as well as in adults working in sheltered workshops [34].

Owing to the heterogeneity in phenotype, severity, and overall impact of ID, the additional effect of co-occurring hearing loss is difficult to quantify. While in typically developing children established developmental trajectories in auditory, language, and social domains allow the impact of hearing loss to be benchmarked against normative data, in individuals with ID, by contrast, the relative contribution of ID and hearing loss to language and behavioral development, independence, and social participation varies widely, and even the onset of hearing loss is often unknown. Yet, in a separate part of our study, we developed a caregiver-reported questionnaire to assess hearing-related quality of life (QoL) in individuals with ID [35]. Multivariable regression analysis showed a small but significant negative association between the degree of hearing loss and hearing-related QoL (β = −0.069; *p* < .001; adjusted R² = .081), adjusted for age, sex, and severity of ID, suggesting that hearing-related QoL may capture the impact of hearing loss and potentially also of hearing care.

### Causes of hearing loss in individuals with ID and associated conditions

The causes of hearing loss in individuals with ID are diverse. Conductive hearing loss may result from chronic otitis media with or without effusion, cerumen impaction, or middle ear malformations. Congenital sensorineural hearing loss—both syndromic and non-syndromic—as well as mixed hearing loss are also common. Many genetic syndromes associated with ID are linked to hearing loss in childhood, while others predispose individuals to early-onset sensorineural hearing loss, such as premature age-related hearing loss [36,37]. Preterm infants born at very low gestational age are at increased risk of both ID and permanent hearing loss, with prevalence rates of the latter ranging from 1.2% to 3% [38,39].

A disproportionately high number of individuals with ID present with obstructive cerumen that completely occludes the ear canal [32]. Removal is often difficult [36] and, in some cases, only possible under general anesthesia. Crandell and Roeser (1993) [40] reported cerumen impaction with associated conductive hearing loss in 28% of adults with ID, compared to only 2–6% in adults without disabilities.

Individuals with Down syndrome, who account for 14–15% of all cases of ID [41], are particularly prone to ear diseases and hearing loss [29,37, 42–48]; between 39% and 70% of them develop hearing loss during their lifetime [49,50]. Comparative studies have shown significantly higher rates of hearing loss in individuals with Down syndrome compared to those with other forms of ID [5,12]: e.g., 73% vs. 22% [51]. Stenotic ear canals and middle ear conditions occur frequently, and presbycusis develops at an earlier age in adults with Down syndrome [52,53] and with other forms of ID [28] than in the general population.

In addition to peripheral hearing loss, individuals with ID commonly experience central auditory processing difficulties [32,54,55] and reduced language comprehension [56], which further complicates communication. The combination of ID and hearing loss, along with auditory and language processing difficulties, is not merely additive but mutually complicating: each condition reduces the individual’s ability to compensate for the other [57–59].

### Barriers to diagnosing and managing hearing loss in individuals with ID

Despite increased epidemiological attention in recent decades, hearing loss in individuals with ID frequently remains undetected and untreated [24,28,32,36,60,61]. At Special Olympics events, up to 4% had undiagnosed severe hearing loss (≥70 dB HL) [30–33]. In two German Special Olympics events, previously-unknown profound hearing loss or deafness was detected in 1.1% [32] and 11.1% [28] of the 1,307 individuals screened. In the latter study, 74% of diagnosed hearing loss cases had not been previously identified.

Hearing loss, often referred to as a “hidden disease” [62], frequently goes unnoticed in individuals with ID—even by close relatives or caregivers. Medical care in this population often prioritizes more apparent or life-threatening issues [57,63], leaving hearing loss overlooked. Certain behavioral or language features may be misattributed to ID when they are actually—or also—related to hearing loss [64,65]. Deaf individuals are also known to exhibit symptoms that mimic autism spectrum disorder, which can further lead to diagnostic confusion [66].

Individuals with ID rarely visit clinical facilities. Even when a hearing loss is diagnosed, follow-up care is often lacking. For instance, only 2% of individuals who received abnormal hearing screening results at Special Olympics events pursued further otological or audiological evaluation and care at a clinical facility, despite having received both oral and written recommendations to do so [54]. Practical and organizational challenges often play a major role here. People with ID almost always require accompaniment by a caregiver, teacher, or parent to attend medical appointments. Those with additional physical conditions may need special transportation. Medical consultations tend to occur only in response to acute complaints. Moreover, individuals with ID may be unable to recognize or articulate health concerns themselves [32].

### Management of ear and hearing conditions in individuals with ID

Ear and hearing disorders in individuals with ID are generally well treatable, especially with early and consistent intervention. Positive outcomes in auditory and speech performance following cochlear implantation in individuals with multiple disabilities, including ID, have been reported [67–69]. A recent systematic review in children [70] demonstrated improvements in environmental sound awareness, non-verbal communication, adaptive skills, QoL, and parental satisfaction, although gains in expressive language typically progress more slowly than in children without disabilities. Another systematic review revealed that cochlear implantation can significantly improve QoL and communication in children with Down syndrome [71]. Individuals with ID also benefit from bone conduction hearing aids and implants [72,73]. Hearing improvement up to normal levels has been demonstrated following consistent medical and surgical treatment of otitis media with effusion in individuals with Down syndrome [47,74]. Hearing care remains feasible and effective even in older adults with ID; after individualized acclimatization training, most individuals over 70 years were able to use hearing aids without difficulty [36]. Speech therapy has likewise been shown to improve speech intelligibility, receptive vocabulary, communicative initiative, and self-reported confidence in individuals with ID, with and without hearing loss [75].

### Programs for regular ear and hearing screening, assessments and intervention

Health screening has been shown to improve the overall health of adults with ID, and randomized studies indicate that simple, low-cost screening instruments can enhance both health and preventive care outcomes in this population [76]. As such, the development and implementation of large-scale, regularly conducted ear and hearing screening and diagnostic programs for individuals with ID represent an important goal for improving healthcare access and outcomes in this underserved group. National and international recommendations for such programs already exist [77–79]. For individuals with Down syndrome, specific clinical guidelines exist, and relevant considerations are also included in broader guidelines [49,52,80,81].

However, a European survey conducted by the Audiology & Intellectual Disability Workgroup of the EFAS (2017a,b) [77,82] revealed that large-scale hearing health programs for individuals with ID are virtually nonexistent across Europe, and that no European country has such a program anchored in national healthcare legislation. Only two countries reported having limited programs in place. Earlier studies on the long-term sustainability of such screening programs have been small in scale and have reported inconsistent outcomes [55,76], partly due to the difficulty of recruiting this specific population for clinical screening initiatives. One major implementation barrier is that individuals with ID rarely visit clinical facilities, as mentioned above. As a result, outreach and low-threshold hearing screening conducted in the natural living environments of people with ID appear to be a more promising approach.

### Aim of the study

This multicenter, partially population-based cohort study, *HörGeist,* investigated the effectiveness, feasibility, and costs of an outreach program involving repeated hearing screening, diagnostics, interventions, and follow-up monitoring for children, adolescents, and adults with ID in their everyday living environments. This program was compared with an invitation-only program including a clinical control cohort and with standard care, defined as the care status at study entry. This report presents a core component of the *HörGeist* study, focusing on the ear and hearing care status of individuals diagnosed with hearing loss before and after participation in the program. We aimed to assess the extent to which the program reduced the proportion of undiagnosed and un- or undertreated hearing loss within the cohort and to identify hinderances to obtaining appropriate care. The overall aim of *HörGeist* is to prepare the implementation of a universal program of regular hearing screening, intervention, and monitoring for individuals with ID in Germany.

## Methods

### Study design

*HörGeist* is a prospective, age-stratified screening study designed to evaluate different approaches to hearing healthcare delivery for individuals with ID. The study compared an outreach hearing detection and intervention strategy (“go strategy”) with two alternatives: (1) standard care, defined as the ear and hearing health status of participants at baseline (T0), which typically reflects unsystematic ad-hoc access to hearing healthcare; and (2) clinic-based screening following written invitations (“come strategy”). In the outreach cohort, the program was conducted in participants’ everyday environments, such as residential facilities, schools, kindergartens, or workplaces. If hearing loss was suspected, follow-up audiological diagnostics, evaluation of any existing interventions, and, where necessary, initiation of new therapeutic measures were carried out—whenever feasible—in the same facility in which the screening had been performed. Structured monitoring followed each intervention.

**Fig 1:**
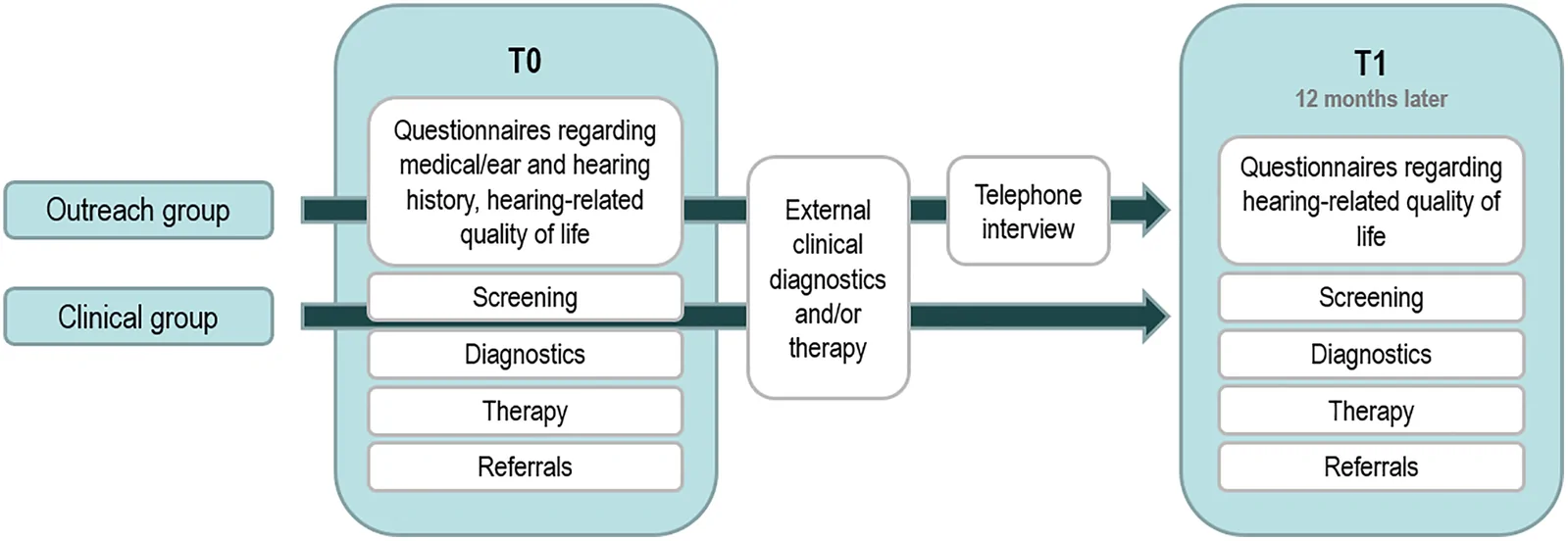
Overall protocol and outcome assessment of the *HörGeist* study (modified from [84]).

In order to evaluate the effectiveness and sustainability of the interventions, the full *HörGeist* screening, diagnostic, monitoring, and intervention protocol was repeated after one year (T1) for each participant (Fig 1). This follow-up aimed to assess diagnostic and treatment outcomes, detect newly developed or transiently resolved hearing loss, and inform recommendations regarding appropriate intervals for future nationwide screening programs in Germany.

Members of the clinical control cohort were invited via their families or legal guardians to undergo the same procedures within a clinical setting. A detailed study protocol has been published by Schwarze et al. (2023) [83].

### Recruitment

The study was conducted in the Rhineland region of North Rhine-Westphalia, Germany. Eligible participants included individuals of all ages with a confirmed diagnosis of ID who were insured by AOK Rhineland/Hamburg, a regional branch of AOK, the largest statutory health insurance provider in Germany and a partner in this project. The only exclusion criterion was if participation in the hearing test would pose a risk to the individual or examiner. The recruitment of participants took place from August 3, 2021 to September 30, 2022.

**Fig 2:**
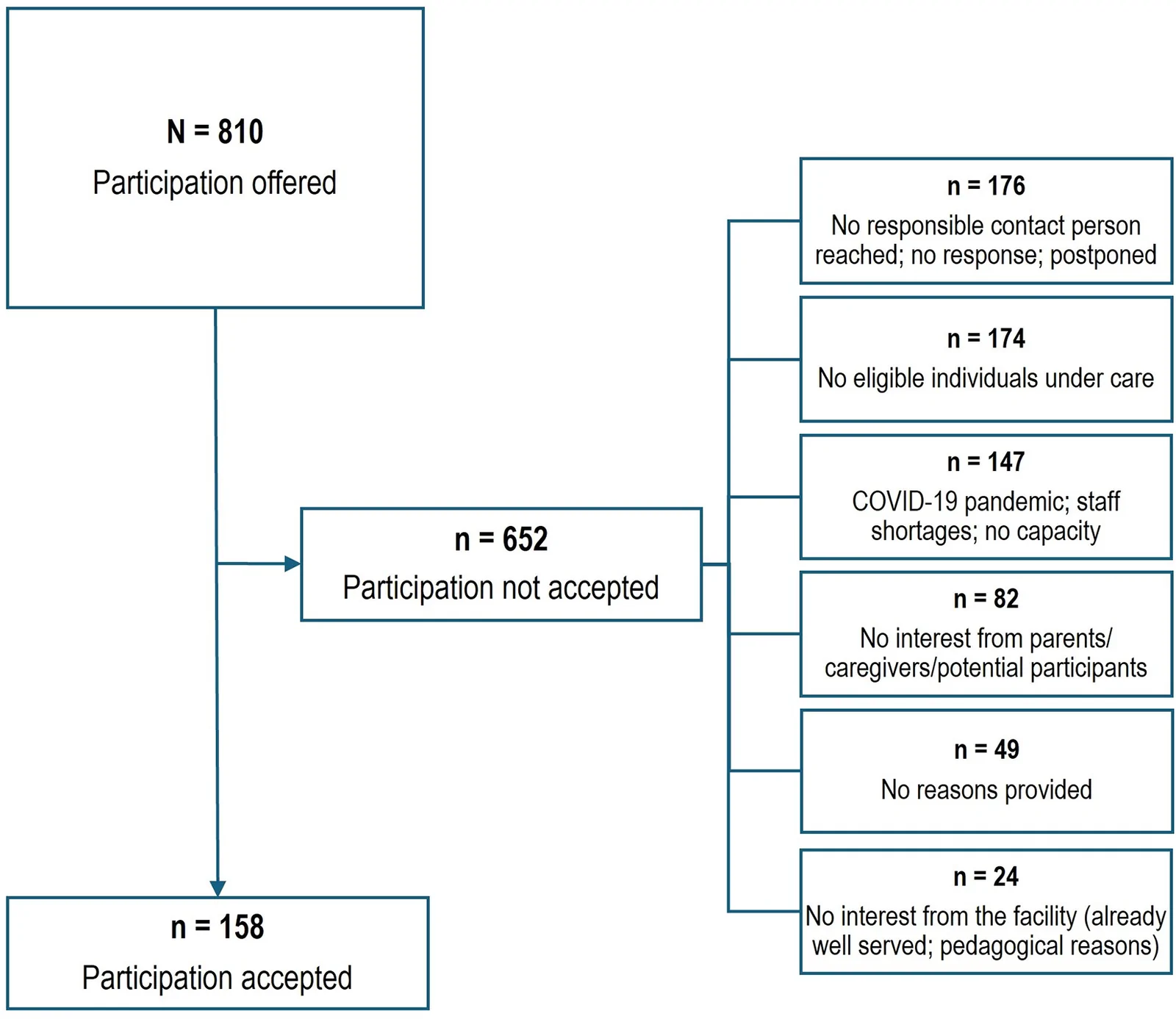
Recruitment outcomes for the outreach cohort and reasons for facility non-participation.

An extensive internet-based search identified 810 institutions in the Rhineland region providing services either exclusively for individuals with ID or offering inclusive settings in which individuals with ID are integrated into mainstream environments. All were invited to participate in the outreach arm of the study and received comprehensive information about the project. Ultimately, 158 facilities enrolled in the study. The main reasons for non-participation were constraints related to the COVID-19 pandemic, as well as limited staff resources and time availability (Fig 2).

Facility staff informed eligible individuals or, where appropriate, their parents or legal guardians about the study. The study team then received the contact details of those who consented to their participation, and appointments for on-site hearing assessments were scheduled in coordination with the respective institutions.

Participants in the clinical control cohort were contacted through postal invitations sent by AOK Rhineland/Hamburg to their families, inviting them to participate in the same procedures at five departments of phoniatrics and pediatric audiology, a German medical specialty addressing communication disorders, including ear and hearing care for individuals with ID. A reminder letter was sent after approximately one year. However, only 12 individuals presented for clinical screening, and none provided informed consent to participate. Consequently, the results reported here pertain exclusively to the outreach cohort.

### Participants

**Fig 3:**
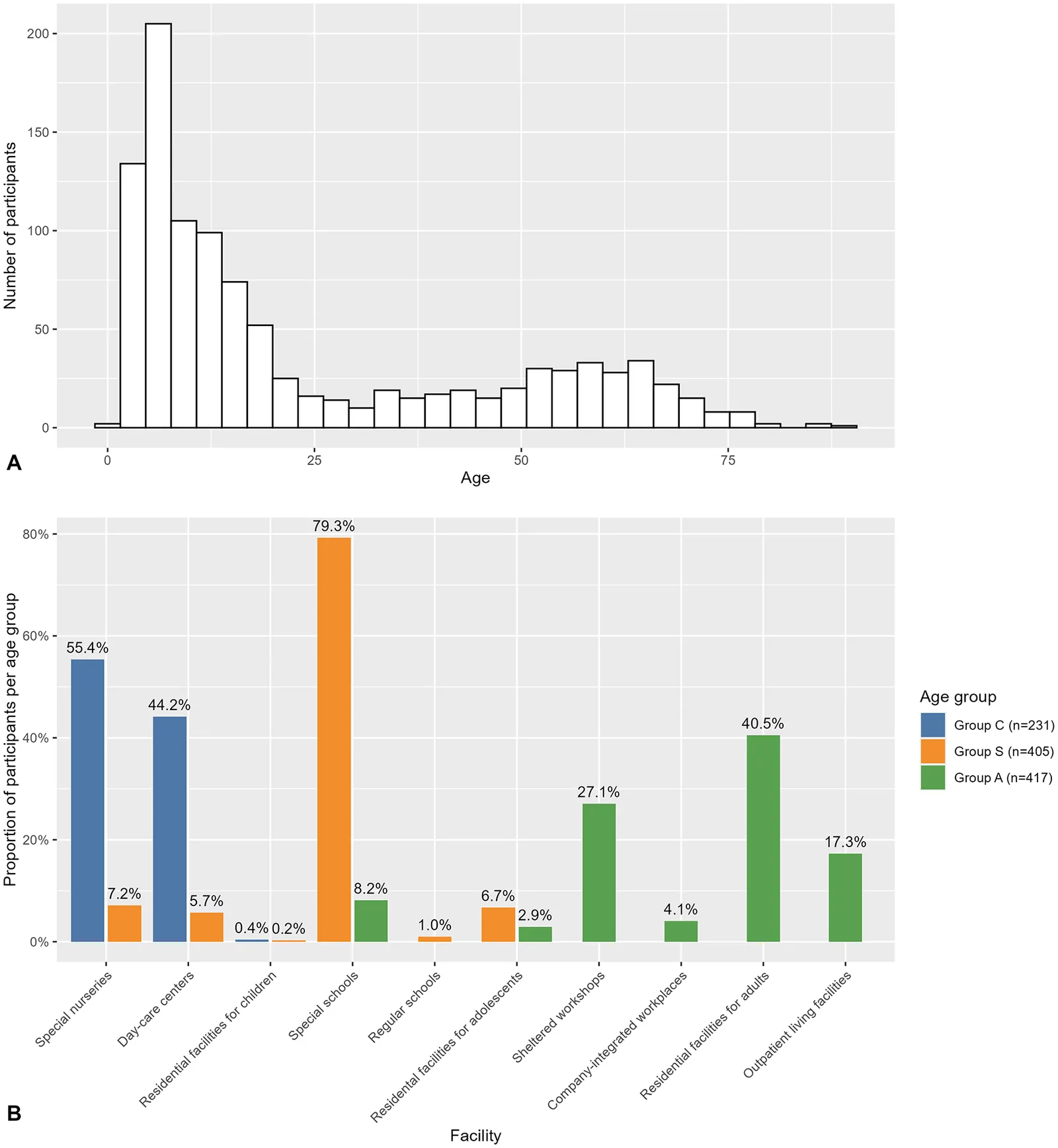
Outreach cohort. Distribution according to (**A**) age and (**B**) screening facility, with percentages representing the distribution within age groups.

In total, 1,194 individuals were enrolled in the study, with 1,053 of them in the outreach program. Among the latter, 662 were male (62.9%), 390 female (37.0%), and one participant identified as intersex. In the control cohort, 88 were male (62.4%) and 53 female (36.6%). The distribution of male and female participants is in line with previous epidemiological findings on individuals with ID [2]. The mean age in the outreach cohort was 23.7 years (range, 1–90 years) and was 22.1 years (range, 2–73 years) in the control cohort (Fig 3A). An overview of the demographics of the outreach cohort is provided in Table 1. Participants of the outreach cohort were stratified into three age groups: young children (0–5 years; group C, n = 231), school-aged children and adolescents (6–17 years; group S, n = 405), and adults (≥18 years; group A, n = 417). The original study design aimed for an even distribution of approximately 350 participants per age group within the outreach cohort. This stratification did not reflect the natural age distribution among individuals with ID but was chosen for methodological and logistical reasons, specifically to demonstrate—using representative samples—that an outreach program could be implemented across all types of age-specific facilities for individuals with ID (Fig 3B), and particularly to reach children, who constitute an especially vulnerable group. The final age distribution deviated from the initial plan, as participants were recruited through their facilities, were age categories only loosely corresponded to the types of facilities attended (e.g., 52 children older than 5 years were still enrolled in daycare or special nursery settings, and 34 individuals older than 18 years still remained in school environments; Table 1).

**Table 1:** Demographic data of the outreach cohort.

Variable	Group C, n = 231^*^	Group S, n = 405^*^	Group A, n = 417^*^	Total, n = 1053^*^
**Age**				
Mean/SD	4/1	10/3	47/17	24/22
Range	1, 5	6, 17	18, 90	1, 90
**Sex**				
Male	144 (62.3%)	278 (68.6%)	240 (57.6%)	662 (62.9%)
Female	86 (37.2%)	127 (31.4%)	177 (42.4%)	390 (37.0%)
Diverse	1 (0.4%)	0 (0%)	0 (0%)	1 (0.1%)
**Facility**				
Special nurseries	128 (55.4%)	29 (7.2%)	0 (0%)	157 (14.9%)
Day care centers with integrative groups or mainstream nurseries/day care centers with individual integration	102 (44.2%)	23 (5.7%)	0 (0%)	125 (11.9%)
Residential facilities for young children	1 (0.4%)	1 (0.2%)	0 (0%)	2 (0.2%)
Special schools	0 (0%)	321 (79.3%)	34 (8.2%)	355 (33.7%)
Mainstream schools with inclusion of children or adolescents with ID	0 (0%)	4 (1.0%)	0 (0%)	4 (0.4%)
Residential facilities for school-aged children and adolescents	0 (0%)	27 (6.7%)	12 (2.9%)	39 (3.7%)
Sheltered workshops	0 (0%)	0 (0%)	113 (27.1%)	113 (10.7%)
Company-integrated workplaces	0 (0%)	0 (0%)	17 (4.1%)	17 (1.6%)
Residential facilities for adults with ID and assisted living facilities	0 (0%)	0 (0%)	169 (40.5%)	169 (16.0%)
Outpatient living facilities	0 (0%)	0 (0%)	72 (17.3%)	72 (6.8%)

* n (%); SD standard deviation

On the basis of our sample size calculation, 141 AOK-insured individuals were age-stratified and invited from the AOK registry to participate as a clinical control cohort.

### Questionnaire

**Fig 4:**
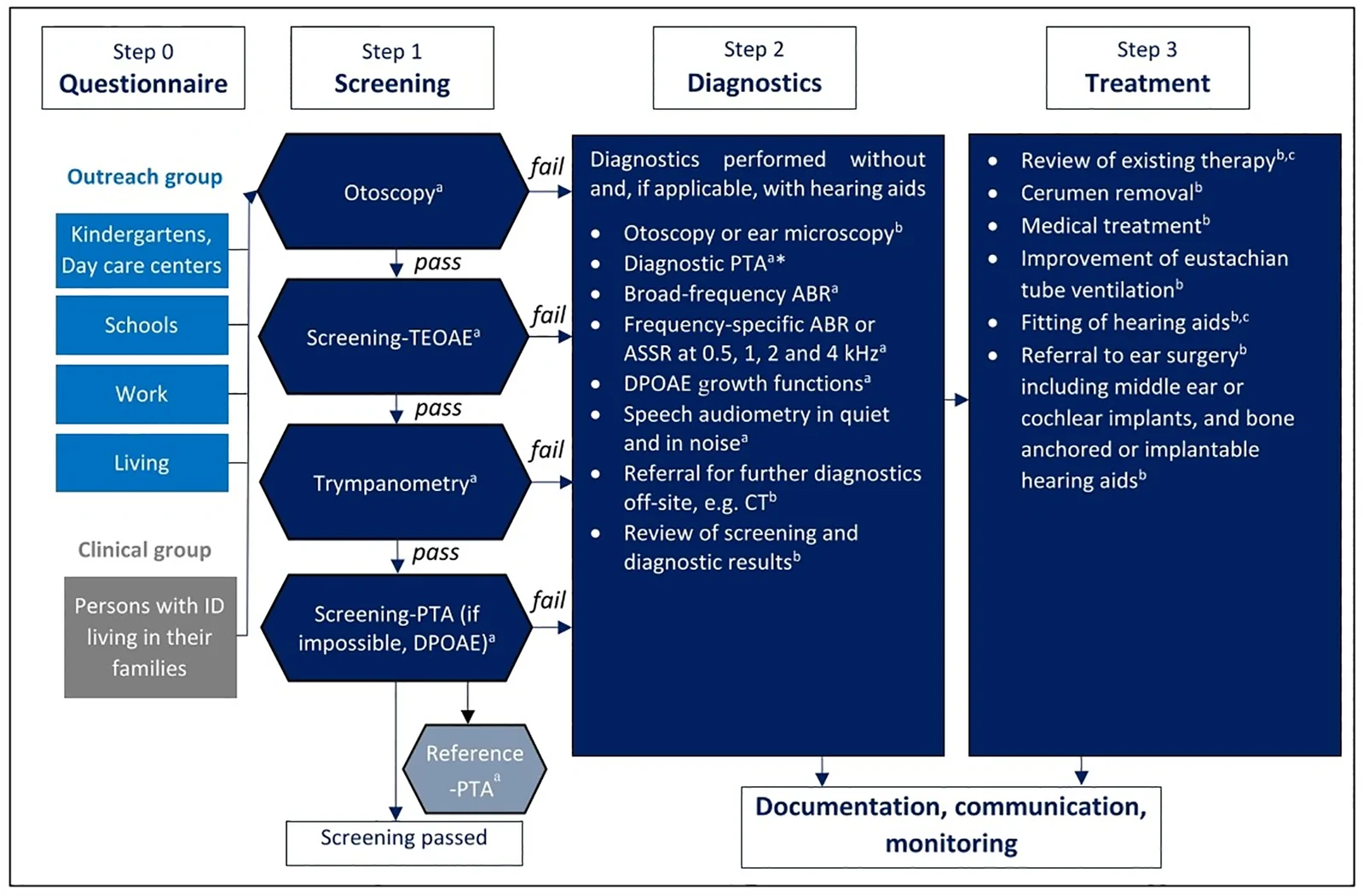
Components of the *HörGeist* screening, diagnostic, and intervention program. ^a^conducted by screening staff; ^b^conducted by physician; ^c^conducted by hearing aid specialist; ^*^In cases of failed screening, reference PTA served as the diagnostic PTA. TEOAE, transient-evoked otoacoustic emissions; PTA, pure-tone audiometry; DPOAE, distortion product otoacoustic emissions; ABR, auditory brainstem response; ASSR, auditory steady-state response; CT, computer tomography (from [35] and modified according to [83]).

Before the initial screening took place, a detailed medical history was taken using a self-developed (the authors are not aware of any comparable tools), structured questionnaire (Fig 4, step 0). For this, caregivers—including family members, residential care staff, physicians, and legal guardians—were contacted by telephone, post, or email. In exceptional circumstances, screening staff completed the questionnaire together with caregivers prior to screening. The questionnaire covered prior diagnosis of hearing loss, history of ear surgery (type, ear, year), hearing device provision (ear, year, type, e.g., hearing aids, cochlear implants, bone conduction systems, middle ear implants, or assistive listening devices), current use (ear), and usage settings (e.g., home, kindergarten/day care center, school, workplace), daily duration of usage, and reasons for non-use in specific settings. The questionnaire also assessed current hearing status, hearing-related quality of life, caregiver-rated degree of ID, comorbidities, current medications, and lifestyle factors [12,35].

### The *HörGeist* protocol

The *HörGeist* program followed a structured, multistep protocol for both the outreach and clinical cohorts. Details are provided in [83] and summarized in Fig 4. All audiometric assessments were performed using the handheld audiometry device *Sentiero* (PATH MEDICAL GmbH). The initial screening phase (Fig 4, Step 1) included video-otoscopy with remote physician review, recording of transient-evoked otoacoustic emissions (TEOAE), tympanometry, and pure-tone audiometry (PTA) at 0.5–8 kHz using either standard or game-based adaptive self-assessment methods (Multiple-choice Auditory Graphic Interactive Check, MAGIC). If PTA was not feasible, distortion product otoacoustic emissions (DPOAE; DPgram at 1–8 kHz) was recorded. Full reference PTA, including air and bone conduction and extended frequencies (0.25 and 6 kHz), served to validate screening results.

Participants who failed any part of the screening were offered on-site diagnostic follow-up (Fig 4, Step 2), including remote video-otoscopy, diagnostic PTA, speech audiometry in quiet and noise (Freiburger monosyllabic test or Mainzer Audiometric Test for Children, MATCH), and, where necessary, auditory brainstem response (ABR) using broadband click or chirp stimuli, and frequency-specific ABR or auditory steady-state response (ASSR). If these standard methods were not feasible or were inconclusive, hearing thresholds were estimated using DPOAE growth functions (DPOAEgf) [34]. All procedures were overseen by the study physician, who reviewed results, conducted remote video-otoscopic examinations where indicated, and provided oral and written follow-up guidance to staff and participants.

On the basis of the diagnostic findings—either from the outreach program itself or from externally conducted assessments—prescriptions for hearing aids, medication, or nasal autoinflation balloons for middle-ear ventilation, as well as referrals for further diagnostic or therapeutic interventions (e.g., cerumen removal, insertion of tympanostomy tubes), were issued. Where hearing loss was diagnosed, therapeutic measures were either offered on-site (such as cerumen removal), or initiated (prescription of hearing aids, medication, or nasal autoinflation balloons), or a referral to external medical care was made (such as for ear surgery or evaluation of candidacy for cochlear implantation) (Fig 4, step 3).

If diagnostic or therapeutic procedures could not be completed on-site, or if caregivers preferred external care, participants were referred for further audiological, otological, radiological, or hearing aid services to external phoniatrics and pediatric audiological, ENT (ear, nose, and throat), or radiological clinics, practices, or hearing aid specialists. Where hearing aids were indicated, caregivers could choose between fitting by a study-affiliated specialist on-site or a local provider. The study physician provided medical summary letters to caregivers, outlining findings and recommendations.

Follow-up on externally conducted diagnostics and interventions was tracked via structured telephone interviews with caregivers between T0 and T1 to monitor the progress and outcomes of these referrals [84]. If telephone contact could not be established, information on implementation was collected during the T1 screening visit. Where hearing aids were prescribed, the program-affiliated hearing-aid specialist contacted participants’ caregivers by telephone to confirm whether they agreed to a hearing-aid fitting and, if so, whether the fitting should be conducted by the program specialist at the facility where the assessment had taken place or by a regional hearing-aid provider.

### Statistics

Descriptive statistics were used in order to determine the proportion of hearing loss diagnosed at study entry and at the end of the study. By definition, hearing loss was assumed if the average hearing threshold, assessed using PTA or MAGIC—or, if these were not feasible, using ABR, ASSR, DPOAEgf, or DPgram—exceeded 20 dB HL across 0.25, 0.5, 1.0, 2.0, 4.0, 6.0, and 8.0 kHz frequencies, or across a subset of these frequencies. This definition is aligned with the WHO classification [85], which defines hearing loss as a PTA average exceeding 20 dB HL across 0.5, 1.0, 2.0, and 4.0 kHz.

The primary outcome variable of the *HörGeist* project was the change in the number of untreated or undertreated cases of hearing loss between T0 and T1, overall and for each age group. This outcome was described using contingency tables and analyzed with Bonferroni-adjusted McNemar tests with Edwards’ correction, applying an overall significance level of 5%.

For other outcome variables, an overall definition of hearing loss incorporating all available information was applied. Hearing loss was assumed if it was identified at T1, as screening and diagnostic assessments at T1, together with interim external diagnostics, yielded a higher proportion of conclusive evaluations of participants’ hearing status than at T0 [86]. If no valid assessment was available at T1 or if participants had dropped out of the study, hearing loss identified at T0 or confirmed through interim external diagnostics and the corresponding telephone interview was accepted. The secondary outcome measures reported here include the number of individuals who received hearing aid prescriptions before T0 and between T0 and T1, the number who underwent ear surgery before T0 and between T0 and T1, and the duration of hearing aid use at home or within the non-private facility at T0 and T1. The practical feasibility of implementing hearing screening, diagnostics, treatment, treatment initiation, and treatment monitoring in the everyday environments of participants in the outreach cohort were also assessed descriptively. This was achieved by reporting the frequency and reasons for missed assessments and interventions at T1.

### Ethics approval and participation consent

The study was approved by the Ethics Committee of the Medical Association of Westphalia-Lippe and the University of Münster (approval number 2020–843-f-S). Prior to participation, all individuals—or, where applicable, their parents or legal guardians—received comprehensive written information about the study. Where appropriate, information was provided in plain, accessible language, supplemented with symbolic images to support participants’ understanding. Written informed consent was obtained from participants or their legal representatives. Caregivers were formally released from their duty of confidentiality in order to enable them to complete the questionnaire and provide relevant supplementary information. The study was conducted in accordance with the Declaration of Helsinki and international Good Clinical Practice guidelines [87,88], and is registered with the German Clinical Trials Register (DRKS00024804). An AI-assisted language editing tool (ChatGPT 5.2, OpenAI) was used solely to improve the clarity and readability of the manuscript. The tool was not used for data analysis, interpretation, or generation of scientific content. All content was reviewed and approved by the authors, who take full responsibility for the manuscript.

## Results

### Ear and hearing conditions, ear surgery and hearing technology used at time of study entry

At study entry (T0), caregivers reported previously or currently known hearing problems in 14.0% of the 1,053 outreach participants. A history of ear surgery was reported in 13.2% participants, and 8.3% had previously received hearing aids, but among these, fewer than half (49.4%) were reported to use them (Table 2).

**Table 2:** Previously known ear and hearing conditions, ear surgery history, and hearing device use at T0 in the outreach cohort.

Variable	Group C n = 231 (%)	Group S n = 405 (%)	Group A n = 417 (%)	Total n=1053 (%)
**Known hearing problems**				
Yes	23 (10.0%)^*^	46 (11.4%)	78 (18.7%)	147 (14.0%)
No	200 (86.6%)	333 (82.2%)	323 (77.5%)	856 (81.3%)
No answer	8 (3.5%)	26 (6.4%)	16 (3.8%)	50 (4.7%)
**Ear surgery**				
Yes	40 (17.3%)	78 (19.3%)	21 (5.0%)	139 (13.2%)
No	180 (77.9%)	288 (71.1%)	363 (87.1%)	831 (78.9%)
No answer	11 (4.8%)	39 (9.6%)	33 (7.9%)	83 (7.9%)
**Hearing devices prescribed**				
Yes	11 (4.8%)	22 (5.4%)	54 (12.9%)	87 (8.3%)
No	211 (91.3%)	360 (88.9%)	347 (83.2%)	918 (87.2%)
No answer	9 (3.9%)	23 (5.7%)	16 (3.8%)	48 (4.6%)
**Of those prescribed: devices used**	n = 11	n = 22	n = 54	n = 87
Yes	5 (45.5%)	10 (45.5%)	28 (51.9%)	43 (49.4%)
No	5 (45.5%)	8 (36.4%)	21 (38.9%)	34 (39.1%)
No answer	1 (9.1%)	4 (18.2%)	5 (9.3%)	10 (11.5%)

### Prevalence of hearing loss identified

Hearing loss was diagnosed in 463 of 1,053 participants (44.0% 12-month prevalence) in the outreach cohort. At baseline (T0), former or current hearing loss had been reported in 147 individuals (14.0%). Of these, the diagnosis was confirmed in 120 cases, ruled out in 24, and remained inconclusive in 3 [35]. Overall, 323 cases (69.8% of all participants with a confirmed hearing loss) represented newly detected, previously undiagnosed hearing loss (missing questionnaire data: n = 20) [35]. Hearing status—and thus potential therapeutic needs—could not be reliably determined in only 35 participants (3.3% of the total sample) [35]. The reasons included refusal of testing and of manipulation of the head, restlessness, wandering around, and loud vocalizations.

Nine hundred seventy-two participants (92.3%) completed both the baseline (T0) and follow-up (T1) assessments [35]. Among the 81 participants who dropped out of the study (7.7% of the outreach sample), 23 (28.4%) had received a prescription or referral for therapeutic action (five hearing-aid prescriptions and 18 therapeutic referrals). Follow-up information was available for 15 of these individuals: two died after receiving hearing aids but before T1; six declined hearing-aid use; in one participant, previously owned hearing aids were optimized, while two declined optimization; two others died before optimization could take place; one participant received topical treatment for external-ear eczema; and in one participant, hearing normalized at clinical follow-up. The proportion of individuals for whom therapeutic action was initiated did not differ markedly between participants who dropped out and the overall sample. However, an additional 26 participants who dropped out (32.1% of all dropouts) had received referrals for further diagnostic evaluation, which also could have resulted in therapeutic interventions.

### Hearing loss related to age, sex, and the severity of intellectual disability

An exploratory logistic regression analysis (without correction for multiplicity) examining the association of hearing loss with age, sex, and severity of ID (categorized as mild, moderate, or severe) showed that age was associated with hearing loss occurrence (adjusted odds ratio [aOR] 1.04 per year, *p* < .001). Higher severity of ID was also associated with an increased risk of hearing loss (moderate vs mild ID: aOR 1.71, *p* = .004; severe vs mild ID: aOR 2.67, *p* < .001), consistent with previous studies [89].

### Treatment initiation and implementation

After initial on-site screening and diagnostics, further diagnostic or therapeutic measures were initiated, prescribed, or recommended for 439 (41.7%) of the 1,053 individuals of the outreach cohort. Of these, 283 participants (26.9%) received prescriptions or referrals for treatment, and 262 (24.9%) were referred to external medical specialists for further diagnostic evaluation and/or subsequent treatment [84]. There was some overlap, as some participants received more than one referral or both a prescription and a referral. Among the 153 participants who received prescriptions for hearing aids, 56 also received referrals to other services, for instance where diagnostic assessment of the contralateral ear was incomplete or inconclusive. Referrals to ENT, phoniatric and pediatric audiological, or other audiological services could have involved diagnostic and/or therapeutic measures, and the eventual outcome often could not be determined in advance. Similarly, referrals for radiologic imaging or cochlear implant evaluation did not necessarily result in a subsequent therapeutic intervention.

The most frequently recommended external measure was referral to an ENT practice or clinic for further evaluation or treatment. This was recommended for 239 individuals (22.7% of the total sample), but over half of these recommendations were not followed (129 cases, 12.0% of total sample). Referral to a phoniatric and pediatric audiological institution was given to 144 individuals (13.7%) but two-thirds of these individuals (95 cases, 9.0% of total) did not receive the recommended care. Sometimes multiple referrals were issued, accounting for discrepancies between the number of referrals and the number of participants requiring treatment. Visits to a hearing-aid professional were recommended in 125 cases (12.0%), and advanced imaging (CT of the petrous bone or head MRI) in six.

**Fig 5:**
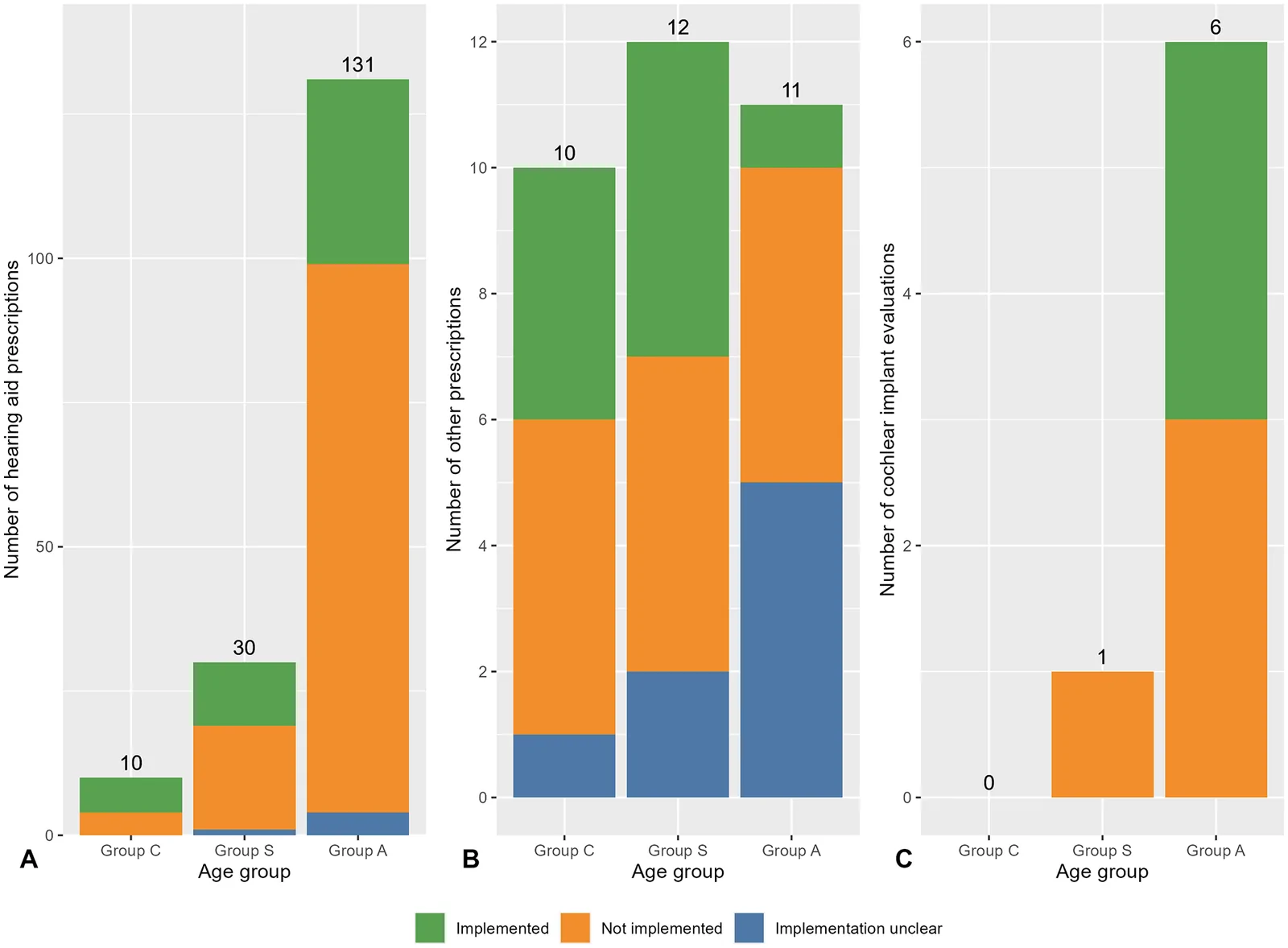
Implementation status of (A) prescribed hearing aids, (B) other prescribed interventions, and (C) the evaluation of cochlear implant candidacy at T1, stratified by age group.

Table 3 summarizes the numbers and proportions of outreach participants for whom therapeutic measures or potentially therapeutic referrals to external medical services were initiated at T0, T1, or in between, whether these measures were implemented, and, where known, the reasons for non-implementation. Fig 5 illustrates the initiation of the most readily traceable therapeutic interventions (hearing aids and other prescriptions) and the implementation status at T1, stratified by age group.

**Table 3:** Initiation of therapeutic measures in the outreach cohort (N = 1053).

Therapeutic measure	Total	Implemented	Implementation unclear	Not implemented	Reasons for non-implementation
**Hearing aid prescription** All At T0 By external diagnostics At T1 Bilateral Unilateral First HA prescription Prescription because HAs too old Prescription because HAs insufficient	171 (16.2%) 153 (14.5%) 12 (1.1%) 6 (0.6%) 162 (15.4%) 9 (0.9%) 148^a^ (14.1%) 6 (0.6%) 17^a^ (1.6%)	49 (4.7%) 37 (3.5%) 12 (1.1%) ND	2 (+ 3 T1) (0.5%) 1 (0.1%) 0 ND	117 (11.1%) 115 (11.0%) 0 1 (0.1%)	23 refusals by caregivers, 33 by participants, 7 by both; 17 did not seek appointment; 1 received no date for appointment; 1 received no medical letter; 31 other reasons; 2 NA 1 refusal by participant, 5 ND
**Other Prescriptions** All At T0 At T1 Medication Cerumen/antimycotic eardrops Antibiotic Nasal autoinflation balloon At T0 At T1 Assistive listening device	33 (3.1%) 32 (3.0%) 1 (0.1%) 6 (0.6%) 5 (0.5%) 1 (0.1%) 25 (2.4%) 1 (0.1%) 1 (0.1%)	10 (0.9%) 10 (0.9%) ND 1 (0.1%)^b^ 1 (0.1%)^b^ 0 9 (0.9%)^d^ ND 0	11 (+ 1 at T1) (1.1%) 11 (1.0%) ND 3 (0.3%) 2^c^ (0.2%) 1 8 (0.8%)^e^ ND 0	11 (1.0%) 11 (1.0%) ND 2 (0.2%) 2 (0.2%) 0 8 (0.8%) ND 1 (0.1%)	2 refusals by caregivers 5 refusals by caregivers, 1 by participant, 1 medical letter not received, 1 NA 1 reason not stated
**Cochlear implant (CI) evaluation**	7 (0.7%)	2 (0.2%), but no CI implanted^f^	0	5 (0.5%)	4 refusals by participants, 1 appointment cancelled by caregiver

^a^1 case: hearing-aid prescription due to insufficient amplification in one ear and first-time hearing-aid provision in the other; ^b^prescription reportedly not received; ENT consultation showed normal ear findings; ^c^ear canal normal at T1; ^d^4 cases treated with nasal autoinflation balloons, 2 with ventilation tubes; 3 cases: normal middle-ear ventilation on external diagnostics; ^e^4 cases: normal middle-ear ventilation at T1, ^f^1 refusal by ENT, 1 refusal by caregiver; HA. hearing aid; ND. not determinable; NA. no data available.

Of the 283 participants for whom treatment was prescribed or recommended at T0, 153 (14.5% of the outreach cohort) received hearing-aid prescriptions, 32 (3.0%) received prescriptions for medication, nasal autoinflation balloons or an assistive listening device, and 98 (9.3%) were referred for cerumen removal, evaluation for hearing-improving surgery or other treatment, with some overlap. Implementation of a prescribed or recommended intervention was documented by telephone interview or at T1 in 119 individuals (49 hearing aids, 4 autoinflation balloons, 18 hearing-improving surgery, and 48 cerumen removals), excluding eight dropouts for whom follow-up was untraceable. Treatment was deemed no longer necessary after external clinical consultation for 18 participants, most commonly due to resolution of middle ear effusion or audiological assessment no longer indicating the necessity of hearing aids.

Forty-three participants were already using hearing aids at T0, of whom 37 continued their use at T1 and six dropped out of the study. Overall, hearing aids were indicated for 171 participants (16.2%) of the outreach cohort (Table 3). Of the 165 participants prescribed hearing aids before T1 and eligible for follow-up, 49 (30%) obtained hearing aids, 115 (70%) did not, and one could not be followed up. Among the 43 participants already using hearing aids at T0, 39 were advised at T0 to have their devices checked or optimized (e.g., fitting adjustments, repairs, new earmolds) and a total of 70 hearing-aid users received this advice at T1.

Further prescriptions for medication, nasal autoinflation balloons, or an assistive listening device with remote microphone technology were issued for 32 participants at T0 and for one participant at T1. Of the 32 participants, 10 (31.2%) either followed the recommendations, received alternative treatment, or were found to have normal hearing status during an ENT or audiological consultation. Of the 24 prescribed nasal autoinflation balloons, four were used; two participants instead underwent tympanostomy tube placement, and three others were documented as having normal middle-ear status at an ENT follow-up. Ear- and hearing-improving surgery was documented in 18 cases (1.7%): tympanostomy tube placement in 14, adenectomy in three, and ear-canal widening in one.

Initial otoscopic findings at T0 indicated that 311 individuals (29.5% of the outreach cohort) had at least one ear canal that was fully or partially occluded by cerumen. On-site cerumen removal was attempted in 130 of these cases. Occlusion was fully or partially resolved in 75 individuals. Occluding cerumen which could not be removed on-site was documented in 91 cases (8.6%) in the medical summary letters and successfully removed by an external service in 48 cases (4.6%).

The most easily traceable interventions were the 197 prescriptions for hearing aids, the assistive listening device, medication, and nasal insufflation balloons issued at T0 (18.7% of the outreach cohort). Of these, 53 (26.9%) adhered to the recommended treatment, two (1.0%) received alternative treatment and 18 (9.1%) consulted an ENT or phoniatric and pediatric audiological service, where treatment was deemed unnecessary (hearing aid fitting in 10 cases, other treatment such as tympanostomy tube placement, middle-ear ventilation balloons, or medication in 8 cases). Recommendations that participants seek treatment were thus reportedly followed in only 73 cases (37.1%), and implementation was declined or remained unclear in 124 (62.9%).

Seven participants (0.7% of the outreach cohort) were considered cochlear implant (CI) candidates due to profound hearing loss or deafness. Only two presented for further specific diagnostics. In one of these cases, the physicians did not agree to implantation without specifying the reason; in the other, the individual declined consent due to anxiety and stress. Radiological examination, which could have resulted in ear surgery, had been recommended for six participants and was undertaken by two of them [84].

### Reduction of un- or undertreated hearing loss

Untreated hearing loss was defined as hearing loss identified at T0, T1, or in the interval between assessments, but not treated at either T0 or T1. Undertreated hearing loss referred to cases in which treatment had been initiated (e.g., hearing aids) but was insufficient, for example because devices were not worn for a sufficient number of hours per day to allow adequate amplification and habituation. In contrast, high-quality care was defined, for example, by whether hearing aid provision effectively brought speech into an adequately audible range and whether devices were worn for a sufficient duration each day. At T0, only six individuals (0.6% of the outreach cohort) met this criterion (Table 4). This situation improved only modestly at T1, where 12 individuals (1.1%) demonstrated high-quality care, indicating persistently high rates of untreated or undertreated hearing loss despite participation in the program.

**Table 4:** Hearing status at T0 and T1 in the outreach cohort (N = 1053).

	Hearing status at T1					
	High-quality treatment received for hearing loss	Un- or undertreated hearing loss	No hearing loss	Unclear hearing status	Data missing	Total
**Hearing status at T0**						
High-quality treatment received for hearing loss	3 (0.3%)	3 (0.3%)	0 (0%)	0 (0%)	0 (0%)	**6 (0.6%)**
Un- or undertreated hearing loss	9 (0.9%)	294 (27.9%)	74 (7.0%)	9 (0.9%)	43 (4.1%)	429 (40.7%)
No hearing loss	0 (0%)	68 (6.5%)	414 (39.3%)	8 (0.8%)	30 (2.8%)	520 (49.4%)
Unclear hearing status	0 (0%)	34 (3.2%)	29 (2.8%)	27 (2.6%)	8 (0.8%)	98 (9.3%)
Total	**12 (1.1%)**	399 (37.9%)	517 (49.1%)	44 (4.2%)	81 (7.7%)	1,053 (100%)

**Fig 6:**
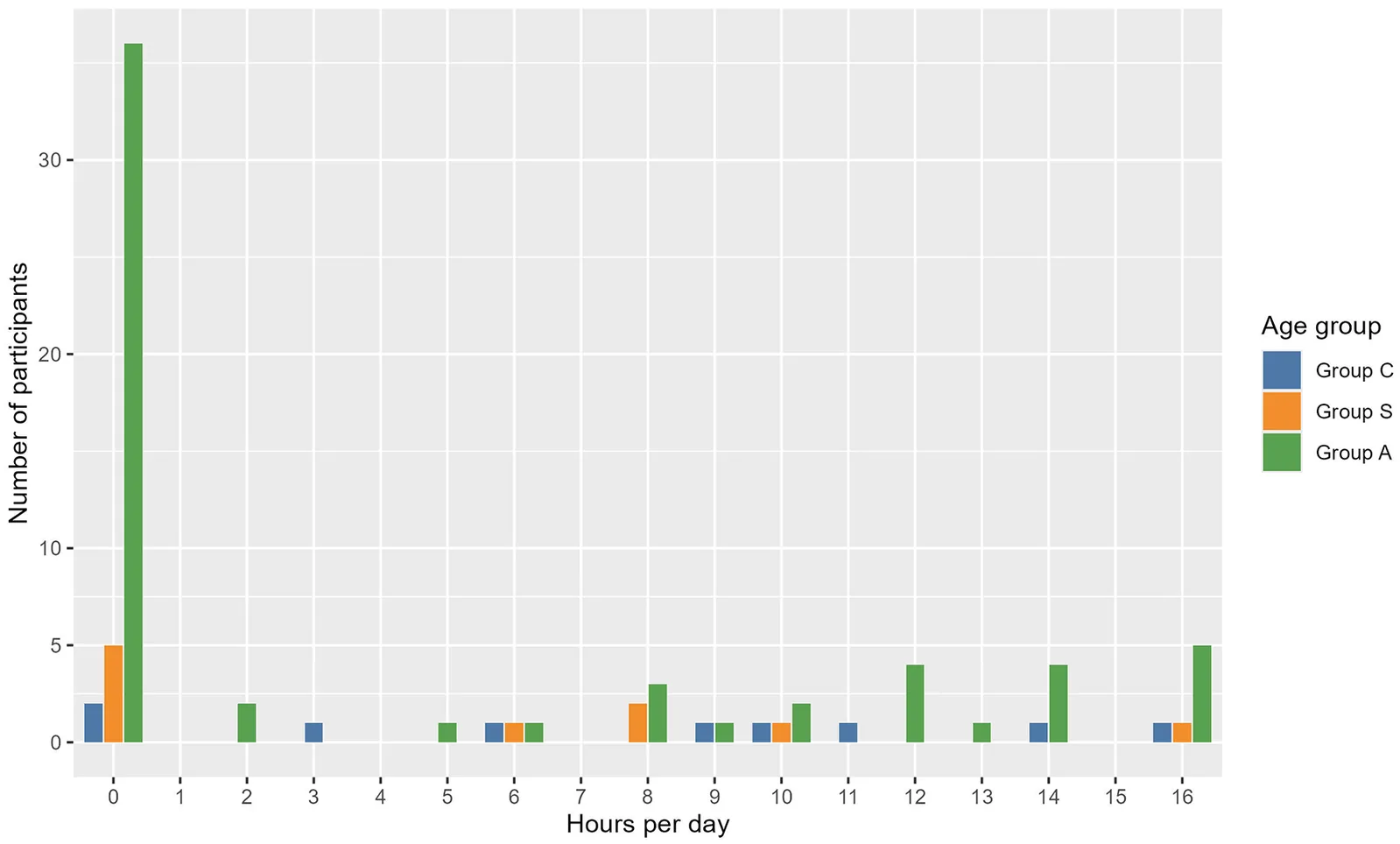
Daily duration of hearing aid use at time point T1, stratified by age group. The histogram includes all 79 participants who were prescribed hearing aids by T1 and for whom data on daily use at T1 were available, including individuals who refused use and 10 for whom hearing aids were deemed unnecessary after external clinical evaluation (this latter introduces some minor bias).

In order to ensure adequate habituation, daily hearing-aid use of at least 8–10 hours is required. In 79 of the 165 individuals who had been prescribed hearing aids before T1 and for whom data on daily use at T1 were available, most either did not use their devices at all or used them for less than the required duration, as demonstrated in the J-shaped distribution in Fig 6; in the latter group, initial use was frequently followed by subsequent rejection of the hearing aids. However, mean and median daily use among the 36 participants with sustained hearing-aid use met the recommended 8–10 hours per day, as shown in Table 5. According to reports from parents, relatives, and caregivers, as documented by the program-affiliated hearing-aid specialist, factors contributing to hearing-aid use included (a) close supervision by parents, educators, and teachers to ensure consistent device use (with approximately similar wearing times at home and in institutional settings for groups C and S, a tendency toward longer use at home in group C, and slightly longer use in school settings in group S), (b) the need to wear hearing aids to follow classroom instruction (group S), (c) the severity of hearing loss—greater severity being associated with a higher likelihood of use (all groups, particularly group A; the longer wearing time in private settings than in institutions in group A underlines that the most and most severe hearing loss among adults occurs in residential care settings, where most are no longer in the workforce [86])—and (d) knowledge of hearing aids among participants, their relatives, and caregivers, as well as their confidence and proficiency in handling them [96].

**Table 5:** Daily duration of hearing aids use in participants with sustained hearing aid use [hours].

	Group C, n = 7	Group S, n = 5	Group A, n = 24	Total, n = 36
Total				
Mean (SD)	9.9 (4.5)	9.6 (3.8)	11.0 (4.3)	10.6 (4.2)
Median (Q1,Q3) Min, Max	10.0 (6.0, 14.0) 3.0, 16.0	8.0 (8.0, 10.0) 6.0, 16.0	12.0 (8.0, 14.0) 2.0, 16.0	10.5 (8.0, 14.0) 2.0, 16.0
In private				
Mean (SD)	5.3 (4.6)	4.0 (6.9)	7.6 (4.5)	6.7 (4.9)
Median (Q1,Q3) Min, Max	4.0 (2.0, 8.0) 0.0, 14.0	0.0 (0.0, 4.0) 0.0, 16.0	7.0 (4.5, 11.0) 1.0, 16.0	6.0 (2.5, 9.0) 0.0, 16.0
At facility (kindergarten/day care center, school, workshop)				
Mean (SD)	4.6 (2.9)	5.6 (3.3)	3.4 (3.7)	3.9 (3.5)
Median (Q1,Q3) Min, Max	6.0 (1.0, 6.0) 0.0, 8.0	6.0 (6.0, 8.0) 0.0, 8.0	1.0 (0.0, 7.5) 0.0, 8.0	5.5 (0.0, 7.5) 0.0, 8.0

### Reasons for not implementing recommended treatment

The most common reasons for not obtaining prescribed hearing aids were caregivers’ or participants’ decisions to decline (30% and 31%, respectively), and failure to initiate appointment scheduling (16%). Additional contributing factors reported include a perceived lack of need for hearing aids, competing health conditions, staff shortages, and limited confidence in hearing aid technology and its use among both caregivers and participants. In 10 cases, local ENT specialists to whom participants were referred did not consider hearing aid provision necessary.

Similar patterns were observed for other recommended treatment: non-implementation was most often attributed to recommendations being declined by caregivers or participants (40%) or non-initiation of appointment scheduling (31%). In six cases, local ENT specialists did not regard the recommended treatment as necessary.

## Discussion

The *HörGeist* project is the first to evaluate the feasibility of a large-scale program of regular hearing assessment, intervention, and monitoring—including optimization of existing treatment—for individuals with intellectual disability, longitudinally and cross-sectionally, in a representative and largely population-based sample (apart from age stratification). An unexpectedly high prevalence of diagnosed hearing loss of 44% supports the need for such an approach [35]. We have demonstrated that an outreach-based model proved substantially more feasible than a clinic-based program, even under non-optimal audiometric conditions [35,86]. Screening validity was high, with a specificity of 96.4% (95% confidence interval [CI] 94.3–97.7) and a sensitivity of 98.0% (95% CI 96.1–99.0) [75], values comparable to those of newborn hearing screening [90]. Screening and diagnostic procedures were reliable and feasible [75]; hearing status could not be determined in only 3.3% of cases [35]. An accompanying economic study highlighted the significant cost impact of hearing loss in ID and showed that improved treatment pathways are needed [91]. However, the therapeutic outcome reported here revealed that only approximately one third (37.1%) of the traceable interventions recommended or prescribed for the hearing losses diagnosed were actually implemented. This is an alarming finding and mirrors the outcome of former cohort studies [27,28,32].

Ensuring access to hearing aids and their sustained use is a key intervention for people with ID and hearing loss; however, in our study, only 30% of hearing-aid prescriptions resulted in effective uptake and use. None of the potential cochlear implant candidates identified received an implant. In only one case was an assistive listening device indicated, though rejected outright. Prescriptions for medication were not redeemed at all, and nasal inflation balloons were used in only 44% of indicated cases, taking into account those in which middle-ear pressure had normalized or ventilation tubes had been inserted.

No ratio between ear operations recommended and performed can be reported because indications for ear surgery were made by external ENT specialists. Furthermore, extraction of occluding cerumen by external clinical services was less well traceable due to its fluctuating status, missing documentation, or poor recall by caregivers, but this condition is of minor importance compared to the aforementioned hearing care necessities.

### Reasons for refusal or non-utilization of recommended treatment

The reasons given for refusing to follow recommended treatment grouped into several key themes. Caregivers or parents and participants often reported that they were already well connected to regional ENT or phoniatric and pediatric audiological services. However, many assumed that the test results obtained were unreliable and questioned the diagnosis of hearing loss. The necessity of hearing aids or medication was frequently doubted and uncertainty and fear regarding the recommended hearing technology or intervention was often expressed.

Refusals were rarely attributable exclusively to either caregivers or participants. Caregivers often act as both advocates and gatekeepers, and participants’ attitudes may mirror those of their caregivers. However, refusal often originated from the participants themselves. Typical caregiver statements included: *“She understands what she needs to,” “He has always been like this; he’s fine,” “She would refuse the hearing aids,”* and *“He would lose the hearing aids.”* Lack of knowledge regarding hearing-aid handling (e.g., insertion, cleaning, drying, battery change), combined with limited time and staff resources, was commonly reported.

The program-affiliated hearing-aid specialist contacted caregivers of all 153 participants with a prescription of hearing aids at T0 in order to offer on-site provision and schedule appointments, often requiring repeated follow-up. Ultimately, on-site hearing-aid fitting occurred in only 24 cases. Additional reasons for non-implementation, beyond those already reported above, included strong aversion to ear-mold impression taking or to ear canal occlusion caused by ear molds, as well as contraindications such as dementia or allergies. In ten cases, local ENTs reported normal hearing and saw no necessity for the hearing aids prescribed, in some instances after cerumen removal without subsequent audiometric confirmation. In other cases, caregivers reported reliance on local providers without follow-through or indicated that internal decisions were pending but then did not respond further despite repeated attempts to contact them.

### Hearing aid fitting and auditory rehabilitation

Even where hearing aids had been fitted, in some cases their use was rejected. Successful adaptation typically begins with low amplification and—as a rule of thumb—a minimum daily wearing time of 8–10 hours is considered necessary to allow the auditory pathway to adapt to the sound, which is often perceived as somewhat artificial initially. Only after this adaptation phase do the devices automatically increase amplification to a level expected to provide sufficient everyday benefit [92,93]. Such increases in amplification to appropriate levels can also be conducted manually by the hearing aid specialist. In many cases, this process did not occur, and only a few participants achieved adequate amplification for soft speech [94].

Hearing rehabilitation can, however, be successful when individuals and their caregivers receive strong professional support [48,68]. Twenty-two participants who received hearing aids from the project’s hearing-aid specialist in their outreach setting were able to be followed through. At 65 dB HL (the sound level of conversational speech), they demonstrated an average benefit of 20.3% [94], compared with 31% reported by one German multicenter study for individuals without ID [95]. However, soft speech was insufficiently amplified according to the median average, and at 50 dB HL virtually no hearing-aid benefit was observed [94]. Maximum speech intelligibility with hearing aids did not reach the 65 dB HL target required by the German assistive device directive and was achieved only at 80 dB HL. The hearing-aid benefit for speech recognition in noise was 14.9% in our participants, compared with 26% in individuals without ID [95]. For these reasons, the number of individuals with hearing loss who we regard as having received high-quality treatment increased only by six between the start and end of the project. Nonetheless, the minimum requirements of the assistive device directive were met in the individuals we analyzed.

In order to inform the development of an appropriate hearing-rehabilitation program and to identify the specific needs of people with ID, we conducted problem-centered interviews in Plain Language with six hearing aid users with ID and analyzed the data using qualitative content analysis [96]. Six staff members from the residential facilities also completed a questionnaire regarding support needs. The responses indicated that overall satisfaction with hearing aids was high. However, substantial needs emerged regarding device handling—from relatively simple tasks such as removing and inserting the hearing aids, and placing them in a drying box, to more complex steps such as cleaning, battery replacement, and functional checks, none of which participants could perform independently. According to staff, none of the participants actively communicated their needs related to managing their hearing loss. These findings indicate a substantial need for post-fitting support for people with ID, particularly regarding independent device handling [96].

To improve the long-term success of hearing aid provision for people with ID, we derived the following conclusions from the hearing aid-related outcomes of the *HörGeist* project [94] and from the above-mentioned pilot study [96]:

- Hearing aid fitting in people with ID can be complex and depends upon the severity of the disability and the individual’s level of compliance; it is therefore essential to use communication that is accessible and tailored to the user.
- One potential advantage of conducting hearing aid fittings in the everyday environment of an individual with ID is that it allows immediate responses to everyday environmental sound and thus enables highly-individualized adjustments.
- Hearing aid fitting within the everyday environment also allows a greater number of caregivers or family members to be involved in the fitting process and to be informed about the importance of consistent hearing-aid use (including agreed-upon wearing time and listening goals) and proper device cleaning; these individuals also need to be trained in the handling and use of hearing aids.
- Although automated hearing-aid features alone may not ensure optimal audibility of soft sounds, clinical experience indicates that combining a well-established fitting approach that ensures sufficient speech audibility with adaptive signal processing is generally effective for people with ID and may allow wearing time to be increased gradually.
- Insertion and removal of hearing aids, as well as battery replacement, should match the person’s individual motor and cognitive abilities.

### Hearing screening in a clinical setting

In the control cohort, potential participants received invitation letters from their health insurance providers—via their families—inviting them to attend a hearing screening in a clinical setting. This recruitment pathway differed substantially from that of the outreach cohort, where institutional leaders were first contacted in writing and subsequently engaged through multiple telephone calls to emphasize the importance of the program, which they then communicated to potential participants or their caregivers. Personal interaction and sustained engagement by recruiting staff therefore likely played a key role in the success of outreach recruitment.

The reasons why only 12 control individuals were presented at clinical facilities—and none consented to study participation—can only be inferred. They most likely reflect well-documented barriers that limit access to healthcare for individuals with intellectual disability. These include the need for a second person (e.g., a caregiver) to schedule and attend appointments, often constrained by limited time and resources; the fact that hearing loss is frequently neither reported by affected individuals nor recognized by caregivers; and the tendency to assign lower priority to hearing health relative to other medical concerns or to assume that ear and hearing care has already been adequately addressed.

### Outlook

As a consequence of our results, we plan to conduct a follow-up study in which we will develop a hearing and communication rehabilitation program for people with ID and their caregivers, drawing on well-validated programs already established for other vulnerable populations [97], implementing it with the participants identified with hearing loss in the current project. The program will draw on educational interventions designed to motivate and counsel individuals with ID and their caregivers, engage primary care physicians, and provide hearing and communication training.

At an international level, our findings suggest that repeated screening and intervention programs generate learning effects among screening personnel, resulting in fewer inconclusive findings (in our study 13% at T0 vs. 6.6% at T1), reduced referrals for external diagnostics (in our study 30% vs. 24%), and improved screening performance and diagnostic confidence. Screening intervals should be short in early childhood, when timely detection and intervention are most critical, particularly in low-resource settings where child health is often prioritized over adults. Intervals should also reflect the age-related increase in hearing loss prevalence, especially among individuals with Down syndrome. We therefore recommend an approach aligned with the EFAS Audiology & Intellectual Disability Workgroup [77], including newborn hearing screening, close follow-up intervals up to six years of age, and more frequent monitoring for individuals with Down syndrome (Table 6). Countries should prioritize the implementation of such programs, alongside accessible rehabilitation strategies that emphasize communication and hearing training in partnership with caregivers.

**Table 6:** Recommendations for audiological screening and management for individuals with intellectual disability, modified from [77].

Individuals with ID in general	Individuals with Down syndrome	Individuals with ID eligible for hearing devices
Neonatal hearing screening		Annual hearing evaluation
Annual screening < age 6	2x/year > age 6	
Every 3 years from age 6–18	Every 2 years from age 6–18	
Every 5 years from age 18–50	Every 3 years from age 18 and 35	
Every 3 years > age 50	Annual > age 35	
Annually if 8h/day or more noise exposure (>80 dBA)		

### Limitations

Some imprecision in determining the need for therapy and the actual uptake of recommended interventions could not be avoided. Recommendations for cerumen removal, the prescription of middle ear inflation balloons, or referral for tympanostomy tube insertion were appropriate at the time but the underlying conditions could have improved spontaneously: previously obstructive cerumen may no longer be present, reduced middle ear pressure may have normalized, and effusion—especially in children—may have resolved. Thus, non-implementation of a therapeutic recommendation does not necessarily indicate a shortcoming in the individual’s care in this study.

Even the prescription of hearing aids, which was sometimes based on a single, potentially uncertain diagnostic assessment, may later prove unnecessary upon follow-up monitoring. In addition, information on treatment provided could not always be obtained. Data from interviews conducted between T0 and T1 were missing in 16 cases because caregivers were unable or unwilling to provide information, could not be contacted, or faced language barriers. Similarly, at T0 and T1, facility caregivers interviewed in kindergartens/daycare centers, schools, or sheltered workshops were sometimes uninformed about treatment or could not be reached.

All of these factors may have led to slight overestimation in the number of interventions required and somewhat underestimated the number of treatments actually undertaken. However, the extent of under- or non-treatment of hearing loss found in this study is comparable to that reported in other studies involving people with ID [27,28,32] and is very large. The uncertainties described in diagnostic assessment and follow-up may be overcome by regularly performed hearing screening and intervention programs.

## Conclusion

In recent decades, the stigmatization and structural disadvantage faced by people with disabilities have increasingly become a focus of societal and political debate. The Convention on the Rights of Persons with Disabilities [98] affirms their right to appropriate medical care and rehabilitation, on an equal basis with persons without disabilities, including early detection and timely treatment of disability-specific health conditions. Against this background, regular hearing screening and intervention for individuals with intellectual disability are essential. We have shown that a coordinated outreach program is feasible and valid, and that medical, technical, and surgical interventions are both effective and necessary. When combined with rehabilitative measures, these interventions can substantially enhance participation and quality of life for people with ID. However, sustainable implementation requires two core elements: (1) broad education of professionals, caregivers, families, and the wider community to raise awareness of hearing problems and the importance of ear and hearing care, and (2) structured auditory rehabilitation and communication training for individuals with confirmed hearing loss, together with their caregivers.
