# Supplementary material for: Overlooked and undertreated: Hearing loss among individuals with intellectual disabilities – A German cohort study
Source: PLOS Glob Public Health. 2026 Aug 19;6(8):e0006872. doi: 10.1371/journal.pgph.0006872 (PMC13489453; doi:10.1371/journal.pgph.0006872)
Supplement: Supplementary file 1 [file pgph.0006872.s002.pdf]

Universitätsklinikum Münster, Malmedyweg 13 48129 Münster

Dr. Catherine Kyobutungi
Editor-in-Chief
PLOS Global Public Health

University Hospital Münster

Dept. of Phoniatrics and Pediatric Audiology

Prof. Katrin Neumann, M.D.
Director

Malmedyweg 13
48149 Münster
Germany

T +49 251 83-56859
F +49 251 83-56889

katrin.neumann@ukmuenster.de
https://www.ukm.de/kliniken/phoniatrie-paedaudiologie

Münster, October 11.02.2026

Dear Dr. Kyobutungi,

Thank you for the opportunity to submit a rebuttal letter regarding our manuscript, which had previously been declined by PLOS Medicine. As requested, we outline below the stated reason for rejection and our response.

The editors of PLOS Medicine prefaced their decision with the journal's general editorial criteria, namely that it seeks to publish research of wide general interest that leads to a substantial advance in clinical management or public health policy, or that provides substantial new insight into disease pathogenesis with a clear path toward clinical application, prioritising conditions contributing to a substantial proportion of the global burden of disease.

The specific reason provided for declining our manuscript was as follows:

"… we do not think it is appropriate for the journal given current editorial criteria and our expectations regarding the extent of conceptual or practical advance that we anticipate being of interest to our broad readership."

We respectfully consider that our study represents a substantial conceptual and practical advance from a global public health perspective.

Persons with intellectual disabilities affect approximately 1.74% of the world's population (GBD 2019 Collaborators 2023). Despite the Convention on the Rights of Persons with Disabilities (United Nations 2006), which affirms the right to appropriate medical care and rehabilitation on an equal basis with persons without disabilities, multiple studies demonstrate persistent healthcare inequities. Individuals with intellectual disabilities experience higher rates of a broad range of diseases (e.g., Carey et al. 2016; Cooper et al. 2025; Kinnear et al. 2018; Sappok et al. 2019), often with earlier onset (Cooper et al. 2015), compared with the general population.

This inequity applies particularly to hearing health. Several studies, including hearing screening initiatives conducted within the Special Olympics (sports competitions and training programmes for persons with intellectual disabilities), have documented a high prevalence of hearing loss (e.g., Willems et al. 2022).

These studies also demonstrated that the majority of affected individuals were either untreated or insufficiently treated, and that even after therapeutic recommendations were issued, clinical follow-up was frequently not realised (Neumann et al. 2009).

In response to these findings, European organisations have issued position statements advocating regular hearing screening for persons with intellectual disabilities, including the Audiology & Intellectual Disability Workgroup of the European Federation of Audiology Societies (2017), Evenhuis (1996), and the NVAVG (Dutch Association of Physicians for People with Intellectual Disabilities, 1995). However, such screening programmes have not been sustainably implemented in any country worldwide; in the Netherlands, screening programmes were introduced only temporarily.

Germany now aims to initiate the first nationwide structured programme combining repeated outreach hearing screening, diagnostic assessment and intervention for persons with intellectual disabilities. The feasibility and outcomes of such a programme were investigated in our population-based cohort study with repeated assessments across all age groups.

Based on comprehensive audiological and otological assessments, the prevalence of hearing loss in our cohort was 44%. When cautiously extrapolated to the global prevalence of intellectual disability (GBD 2019 Collaborators, 2023), this would imply that approximately 0.76% of the world's population may be affected by both intellectual disability and hearing loss—most cases likely remaining undetected and untreated or undertreated.

According to the WHO International Classification of Functioning, Disability and Health (ICF), unrecognised hearing loss in persons with intellectual disabilities represents an additional functional impairment that further restricts communication, social participation, autonomy and quality of life (Zielonkowski et al. 2025). Our study, based on more than 1,000 individuals assessed twice at a one-year interval, demonstrates both the feasibility of implementing an outreach-based screening and diagnostic programme and the substantial unmet need in hearing care across the lifespan.

At the same time, the only modest improvement in care uptake after programme participation clearly indicates that sustainable large-scale implementation requires public and professional awareness, structural integration into healthcare systems and rehabilitation strategies that include hearing and communication training and actively involve persons with intellectual disabilities and their caregivers. This situation parallels the historical implementation of global newborn hearing screening programmes, which likewise required coordinated policy efforts and system-level change before becoming widely implemented.

Given the documented global healthcare inequities affecting persons with intellectual disabilities and ongoing WHO initiatives in Ear and Hearing Care, we believe that our findings are highly relevant to global public health systems. Our study quantifies a largely hidden burden of disease, provides evidence for the feasibility of a structured population-based approach and highlights the systemic requirements for sustainable implementation.

We therefore hope that the editors of PLOS Global Public Health will consider the manuscript within this global health and equity framework, and we would be delighted if it were considered suitable for publication in your journal.

Kind regards,

Katrin Neumann (on behalf of all authors)

References

Audiology & Intellectual Disability Workgroup of the European Federation of Audiology Societies (EFAS). Guideline proposal for audiological hearing screening and management for people with intellectual disability. 2017 [cited 2026 February 2]. Available from: https://efas.ws/wp-content/uploads/2023/03/2017-06-Background-and-Preliminary-Guidelines.pdf

Carey IM, Shah SM, Hosking FJ, DeWilde S, Harris T, Beighton C, et al. Health characteristics and consultation patterns of people with intellectual disability: a cross-sectional database study in English general practice. Br J Gen Pract. 2016 Apr;66(645):e264–70. doi: 10.3399/bjgp16X684301.

Cooper SA, McLean G, Guthrie B, McConnachie A, Mercer S, Sullivan F, et al. Multiple physical and mental health comorbidity in adults with intellectual disabilities: population-based cross-sectional analysis. BMC Fam Pract. 2015 Aug 27;16:110. doi: 10.1186/s12875-015-0329-3.

Emerson E, Hatton C, Baines S, Robertson J. The physical health of British adults with intellectual disability: cross sectional study. Int J Equity Health. 2016 Jan 20;15:11. doi: 10.1186/s12939-016-0296-x.

Evenhuis HM. Dutch consensus on diagnosis and treatment of hearing impairment in children and adults with intellectual disability. The Consensus Committee. J Intellect Disabil Res. 1996 Oct;40 (Pt 5):451-6. doi: 10.1046/j.1365-2788.1996.788788.x.

Kinnear D, Morrison J, Allan L, Henderson A, Smiley E, Cooper SA. Prevalence of physical conditions and multimorbidity in a cohort of adults with intellectual disabilities with and without Down syndrome: cross-sectional study. BMJ Open. 2018 Feb 5;8(2):e018292. doi: 10.1136/bmjopen-2017-018292.

Neumann K, Hey C, Baumann U, Montgomery J, Euler HA, Hild U. Eine hohe Prävalenz von Hörstörungen bei den Special Olympics belegt die Notwendigkeit von Hörscreenings für Personen mit geistiger Behinderung. [German] [A high prevalence of hearing loss among Special Olympics participants highlights the need for hearing screenings in individuals with intellectual disabilities.] In: Wegner M, Schulke HJ, editors. Kieler Schriften zur Sportwissenschaft. Kiel: Sport-Thieme GmbH; 2009. pp. 6–17.

NVAVG (Nederlandse Vereniging voor Artsen voor Verstandelijk Gehandicapten). Richtlijnen voor diagnostiek en behandeling van slechthorendheid bij verstandelijk gehandicapten. [Dutch] [Guidelines for diagnostics and treament of hearing loss in peopleperson with an intellectual disability]. 1995 [cited 2025 Jul 1]. Available from: http://nvavg.nl/wp-content/uploads/2014/upload/standaarden/standaard---1995-02-slechthorendheid.pdf

Sappok T, Diefenbacher A, Winterholler M. The medical care of people with intellectual disability. Dtsch Arztebl Int. 2019 Nov 29;116(48):809–16. doi: 10.3238/arztebl.2019.0809.

Willems M, Acke F, Lannon B, Leyssens L, Maes L, Marks L. Global Data on Ear and Hearing Screening in an Intellectual Disability Population. Am J Intellect Dev Disabil. 2022 Mar 1;127(2):125-134. doi: 10.1352/1944-7558-127.2.125.

United Nations. Convention on the Rights of Persons with Disabilities. 2006, [cited 2025 Nov 17]. Available from: https://www.ohchr.org/en/instruments-mechanisms/instruments/convention-rights-persons-disabilities
