# Supplementary material for: Overlooked and undertreated: Hearing loss among individuals with intellectual disabilities – A German cohort study
Source: PLOS Glob Public Health. 2026 Aug 19;6(8):e0006872. doi: 10.1371/journal.pgph.0006872 (PMC13489453; doi:10.1371/journal.pgph.0006872)
Supplement: Supplementary file 2 [file pgph.0006872.s003.docx]

Possible table approach

| Anticipated needed outcome | Known hearing needs  n=147 (14%) | Those with newly confirmed atypical hearing thresholds at T0 n=323 (69.8%)  Recommended intervention for 439 (41.7%) | Outcome at T1  Including Lost to follow-up (LTFU) |
| --- | --- | --- | --- |
| Referral to ENT |  | 22% (n=239/1053) | 54% LTFU (n=129/239)  18 resolved issue |
| Referral to audiology |  | 25% (n=114/439) | 83% LTFU (n=95/114) |
| Cerumen removal |  | 70% (n=311/439)  29.5% (n=311/1053) | (n=48/?) |
| Antibiotic |  |  |  |
| NAB |  | 5% (n=25/439) | 80% did not use (n=16/20)  2 with PE tubes  3 resolved |
| Ear surgery | 13.9 |  |  |
| Type of hearing thresholds  Severe to profound  Unable to determine |  | 2.8% (n=9/323)  3.3% (n=35/1053) |  |
| Prescribed Amplification/suggested | 8.7% (n=87) | 45% (n=148/323)  Narrative said 153 |  |
| Using amplification | 4.3% (n=43) | Updates required  53% (n=23) |  |
| Therapy suggested |  | 26.9% (n=283/439) |  |
