# Supplementary material for: Overlooked and undertreated: Hearing loss among individuals with intellectual disabilities – A German cohort study
Source: PLOS Glob Public Health. 2026 Aug 19;6(8):e0006872. doi: 10.1371/journal.pgph.0006872 (PMC13489453; doi:10.1371/journal.pgph.0006872)
Supplement: Supplementary file 3 [file pgph.0006872.s004.docx]

**Response to Reviewers**

Regarding manuscript PGPH-D-26-00483

“Overlooked and undertreated: hearing loss among individuals with intellectual disabilities – a German cohort study”

Dear Dr. Edwards, dear Associate Editor and Reviewers,

We would like to thank you and the reviewers for the thoughtful and constructive comments on our manuscript "Clinical diagnostics are rarely conducted after people with intellectual disabilities fail hearing screening". The reviewers clearly approached the manuscript with great care and expertise, and we are grateful for the time and effort invested in addressing all relevant issues and uncertainties.

We would like to address the comments as follows; the manuscript has also been revised accordingly.

Dear Dr. Neumann,

Thank you for submitting your manuscript to PLOS Global Public Health. After careful consideration, we feel that it has merit but does not fully meet PLOS Global Public Health’s publication criteria as it currently stands. Therefore, we invite you to submit a revised version of the manuscript that addresses the points raised during the review process.

The manuscript has been evaluated by two reviewers, and their comments are available below. The reviewers have raised a number of major concerns. They request some language revision to avoid implicit bias, as well as updates to the literature review. They also request clarification in a number of areas and updates to ensure the reporting of results in the text is in line with that presented in figures and tables. Could you please carefully revise the manuscript to address all comments raised?

Please submit your revised manuscript by May 17 2026 11:59PM. If you will need more time than this to complete your revisions, please reply to this message or contact the journal office at [globalpubhealth@plos.org](mailto:globalpubhealth@plos.org). When you're ready to submit your revision, log on to <https://www.editorialmanager.com/pgph/> and select the 'Submissions Needing Revision' folder to locate your manuscript file.

Please include the following items when submitting your revised manuscript:

- A letter that responds to each point raised by the editor and reviewer(s). You should upload this letter as a separate file labeled 'Response to Reviewers'.
- A marked-up copy of your manuscript that highlights changes made to the original version. You should upload this as a separate file labeled 'Revised Manuscript with Track Changes'.
- An unmarked version of your revised paper without tracked changes. You should upload this as a separate file labeled 'Manuscript'.

Guidelines for resubmitting your figure files are available below the reviewer comments at the end of this letter.

As the corresponding author, your ORCID iD is verified in the submission system and will appear in the published article. PLOS supports the use of ORCID, and we encourage all coauthors to register for an ORCID iD and use it as well. Please encourage your coauthors to verify their ORCID iD within the submission system before final acceptance, as unverified ORCID iDs will not appear in the published article. Only the individual author can complete the verification step; PLOS staff cannot verify ORCID iDs on behalf of authors.

We look forward to receiving your revised manuscript.

Kind regards,

Jen Edwards
Staff Editor
PLOS Global Public Health

Journal Requirements:

1. Please provide a detailed online Financial Disclosure statement. This is published with the article. It must therefore be completed in full sentences and contain the exact wording you wish to be published.

a) State the initials, alongside each funding source, of each author to receive each grant. For example: “This work was supported by the National Institutes of Health (####### to AM; ###### to CJ) and the National Science Foundation (###### to AM).”

b) State what role the funders took in the study. If the funders had no role in your study, please state: “The funders had no role in study design, data collection and analysis, decision to publish, or preparation of the manuscript.”

For more information, please go to our submission guidelines:
<https://journals.plos.org/globalpublichealth/s/submission-guidelines#loc-financial-disclosure-statement>

2. Please ensure that the funders and grant numbers match between the Financial Disclosure field and the Funding Information tab in your submission form. Note that the funders must be provided in the same order in both places as well.

3. When completing the data availability statement of the submission form, you indicated that you will make your data available on acceptance. We strongly recommend all authors decide on a data sharing plan before acceptance, as the process can be lengthy and hold up publication timelines. Please note that, though access restrictions are acceptable now, your entire data will need to be made freely accessible if your manuscript is accepted for publication. This policy applies to all data except where public deposition would breach compliance with the protocol approved by your research ethics board. If you are unable to adhere to our open data policy, please kindly revise your statement to explain your reasoning and we will seek the editor's input on an exemption. Please be assured that, once you have provided your new statement, the assessment of your exemption will not hold up the peer review process.

For further assistance, you may go to: <http://journals.plos.org/globalpublichealth/s/data-availability>

**Reply**: After extensive discussions within our biomathematical project team, which is responsible for data security, and in consultation with Dr. Jay-ar Formentera Medes from PLOS Global Public Health, we sought to identify a feasible solution for data sharing. As a result, we have now included the following explanation in the data availability statement:

“Dear Dr. Jay-ar Formentera Medes,

We are writing to you regarding the issue of data availability connected to the aforementioned publication. We share the view that transparency in science is of the utmost importance and that, wherever possible, research data should be shared. Therefore, over the past few weeks, we have held intensive discussions with the individuals responsible for data protection in the project and explored various approaches for granting other scientists access to the data collected in our research project. Our proposals included, for example, sharing the raw data, or allowing other scientists to write analysis code, which would then be executed at our institution before the results were sent back to the scientists. We also inquired whether it would be possible to make aggregated data publicly available. Unfortunately, all these solutions were rejected by the data protection officers, citing the relatively restrictive consent form provided by the patients. Due to the large number of participants, it is unfortunately also not possible to obtain expanded consent retroactively that would allow for a more liberal publication of patient data. For our current and future projects, we will focus on designing the consent form in a way that allows for more liberal data sharing.

We were informed that, for our project, any data processing not directly related to the study is prohibited. However, we were able to reach a consensus that both the journal and other researchers should be able to validate our findings. Since the informed consent form explicitly prohibits the release of data to third parties, we can offer to allow researchers who wish to validate the results to write code for the analysis, which will then be executed on our computers. The results can then be made available to the inquiring researchers. To facilitate this, we would provide a dataset description as well as sample data which could then be used to write the code. We have been instructed to note that even in these analyses, no analyses may be conducted that go beyond the research questions addressed in the study; only the validation of our results is permitted. However, this approach at least ensures that the results we have obtained can be validated by the scientific community.

We hope that you will be able to accept the method of accessing the data outlined above, and we are, of course, also available for a personal discussion, for example, by phone or video call. It is very important to us that our manuscript be published in PLOS Global Public Health, also given the significant amount of work that has already been invested by both the reviewers and ourselves.

Sincerely,

Martin Scharpenberg (on behalf of the authors)

Dr. Martin Scharpenberg

Projektverantwortung

Universität Bremen

Kompetenzzentrum für Klinische Studien Bremen

Fachbereich 03 I Mathematik und Informatik

Raum 41120

Linzer Straße 4

28359 Bremen

Tel. 0421 218-63789

Fax 0421 218-9863789

mscharpenberg@uni-bremen.de

www.kksb.uni-bremen.de

www.uni-bremen.de.”

4. If the reviewer comments include a recommendation to cite specific previously published works, please review and evaluate these publications to determine whether they are relevant and should be cited. There is no requirement to cite these works unless the editor has indicated otherwise.

Additional Editor Comments (if provided):


[Note: HTML markup is below. Please do not edit.]

Reviewers' comments:

Reviewer's Responses to Questions

**Comments to the Author**

1. Does this manuscript meet PLOS Global Public Health’s [publication criteria](https://track.editorialmanager.com/CL0/https:%2F%2Fjournals.plos.org%2Fglobalpublichealth%2Fs%2Fcriteria-for-publication/1/010f019d616b7b50-d99fae09-5dca-4ab6-ae70-24180f897a79-000000/GzYmRDCHo3kcFsVfp1lxCx6H9MINUq9E4C669unus3c=257)? Is the manuscript technically sound, and do the data support the conclusions? The manuscript must describe methodologically and ethically rigorous research with conclusions that are appropriately drawn based on the data presented.

Reviewer #1: Yes

Reviewer #2: Yes

2. Has the statistical analysis been performed appropriately and rigorously?

Reviewer #1: N/A

Reviewer #2: Yes

3. Have the authors made all data underlying the findings in their manuscript fully available (please refer to the Data Availability Statement at the start of the manuscript PDF file)?

The [PLOS Data policy](https://track.editorialmanager.com/CL0/https:%2F%2Fjournals.plos.org%2Fglobalpublichealth%2Fs%2Fdata-availability/1/010f019d616b7b50-d99fae09-5dca-4ab6-ae70-24180f897a79-000000/zB50yJG7WAZlmVJCbWSsItczPG5SUJ99isXCSFaHFyY=257) requires authors to make all data underlying the findings described in their manuscript fully available without restriction, with rare exception. The data should be provided as part of the manuscript or its supporting information, or deposited to a public repository. For example, in addition to summary statistics, the data points behind means, medians and variance measures should be available. If there are restrictions on publicly sharing data—e.g. participant privacy or use of data from a third party—those must be specified.

Reviewer #1: Yes

Reviewer #2: Yes

4. Is the manuscript presented in an intelligible fashion and written in standard English?

PLOS Global Public Health does not copyedit accepted manuscripts, so the language in submitted articles must be clear, correct, and unambiguous. Any typographical or grammatical errors should be corrected at revision, so please note any specific errors here.

Reviewer #1: Yes

Reviewer #2: Yes

5. Review Comments to the Author

Please use the space provided to explain your answers to the questions above. You may also include additional comments for the author, including concerns about dual publication, research ethics, or publication ethics. (Please upload your review as an attachment if it exceeds 20,000 characters)

**Response to comments of the reviewers:**

**Reviewer #1:** see uploaded document example of table.

**Comment 1:** The paper, Overlooked and undertreated, hearing loss among individual with IDD is an important contribution to the literature. There is very little published on rates of abnormal hearing thresholds among adults with IDD.

The authors have developed an important program across Germany that provides strong guidance on the unmet health care needs of children and adults with intellectual disability.

**Reply:** We thank the reviewer for this positive assessment and agree that the topic—improving ear and hearing health among individuals with intellectual disabilities—has been relatively little studied and reported. We strongly hope that this manuscript will support the nationwide implementation of the described outreach program for ear and hearing care for people with intellectual disabilities in Germany and that it may serve as an international model for regular hearing screening, diagnostics, interventions, and follow-up monitoring, thereby contributing to the fulfilment of the UN Convention on the Rights of Persons with Disabilities.

**Comment 2:** This paper would benefit from streamlining a bit within the background section and results as it is lengthy and sometimes with quite detailed. For example in the background, causes of hearing differences among individuals with ID, could be more concise. If there is literature on the impact of hearing differences in IDD that would also provide more impetus for the reader to want to follow hearing and outer and middle ear issues. It is possible that within this journal, the background should be expansive. I am more accustomed to medical writing where the discussion is the section with most in-depth information.

**Reply:** We have revised the manuscript with this perspective in mind and have streamlined both the Introduction and the Results where possible. At the same time, our original aim was to present a comprehensive paper that makes the issue of ear and hearing health in individuals with ID—and its inherent complexity—accessible to a broad readership that may not be familiar with the topic. For this reason, we selected PLOS Global Public Health, which does not impose strict word limits. We have taken care to preserve this objective while implementing the requested reductions.

In addition, the reviewer suggested substantial additions, including more recent literature, additional figures, and new data analyses. While we agree that these revisions strengthen the manuscript, they have also contributed to a slight overall increase in length despite our efforts to condense the text.

The mentioned complexity also explains why the reviewer’s suggestion—“If there is literature on the impact of hearing differences in IDD, that would also provide more impetus for the reader to engage with hearing and outer and middle ear issues”—is difficult to address. Owing to the heterogeneity in phenotype, severity, and overall impact of intellectual disability itself, the additional impact of co-occurring hearing loss is challenging to quantify. For example, in typically developing children, well-established developmental trajectories and milestones for auditory, language, and social development allow for benchmarking the effects of hearing loss against normative data. In contrast, for individuals with ID, the extent to which intellectual disability versus hearing loss contributes to impairments in language and behavioral development, independence, and social participation remains highly individual. In many cases, even the timing of onset of hearing loss is unknown.

One robust indicator, however, is hearing-related quality of life. We have not only developed an instrument to assess this construct but have also demonstrated that, according to caregiver ratings, it decreases significantly with increasing severity of hearing loss—even when caregivers were unaware of the degree of hearing loss (Zielonkowski et al. 2025).

We have therefore inserted the following passage in the Introduction section:

“Owing to the heterogeneity in phenotype, severity, and overall impact of ID, the additional effect of co-occurring hearing loss is difficult to quantify. While in typically developing children, established developmental trajectories in auditory, language, and social domains allow the impact of hearing loss to be benchmarked against normative data, in individuals with ID, by contrast, the relative contribution of ID and hearing loss to language and behavioral development, independence, and social participation varies widely, and even the onset of hearing loss is often unknown. Yet, in a separate part of our study, we developed a caregiver-reported questionnaire to assess hearing-related quality of life (QoL) in individuals with ID [35]. Multivariable regression analysis showed a small but significant negative association between the degree of hearing loss and hearing-related QoL (β = −0.069; *p* < .001; adjusted R² = .081), adjusted for age, sex, and severity of ID, suggesting that hearing-related QoL may capture the impact of hearing loss and potentially also of hearing care.”

**Comment 3:** While I can see considerable work and depth of their literature review, some references were quite old and there may be newer publications that could be presented instead. There were also publications that were described as “forthcoming”. I am also not familiar with this approach. If a paper is in press or epub ahead of print, that makes sense, but a publication in preparation would typically be described as a communication with the authors. One example of a citation as forthcoming was #72. I think at the time of manuscript preparation this was e pub ahead (as of January 2026) and is now published as of April 2026. Another example of an older citation is the AAP guidelines for the medical care of Down syndrome. This has been updated as of 2022 and there is now an adult healthcare guidelines for individuals with Trisomy 21. There is also now a JCIH statement as of 2019 which is not pubmed queriable (JEHDI). Some of the cochlear implant citations are old (performance of multiply handicapped children using CI’s.

**Reply:** We thank the reviewer for this valuable suggestion and agree with the point raised. We have now implemented additional recent literature listed below (which has slightly increased the length of the manuscript). Overall, this literature demonstrates positive hearing, language, and communication outcomes following the treatment of hearing loss in individuals with ID using medication, hearing aids, cochlear implants, and other otologic surgeries. While quality of life as reported by parents or other caregivers is generally rated positively, language outcomes appear to depend more strongly on the severity of ID.

Added literature:
Bull MJ, Trotter T, Santoro SL, Christensen C, Grout RW; COUNCIL ON GENETICS, et al. Health supervision for children and adolescents with Down syndrome. Pediatrics. 2022 May 1;149(5):e2022057010. doi: 10.1542/peds.2022-057010.

Butcher E, Dezateux C, Cortina-Borja M, Knowles RL. Prevalence of permanent childhood hearing loss detected at the universal newborn hearing screen: systematic review and meta-analysis. PLoS One. 2019 Jul 11;14(7):e0219600. doi: 10.1371/journal.pone.0219600.

Bee-See G, Zulkefli NAM, Abdullah A, Umat C, Nor NK, Ismail J, O'Leary SJ. Cochlear implantation outcomes in children with multiple disabilities: a topic that's worth revisiting. Braz J Otorhinolaryngol. 2024 Jul-Aug;90(4):101423. doi: 10.1016/j.bjorl.2024.101423.

Caragli V, Monzani D, Genovese E, Palma S, Persico AM. Cochlear implantation in children with additional disabilities: a systematic review. Children (Basel). 2023 Oct 5;10(10):1653. doi: 10.3390/children10101653.

Clarós P, Remjasz A, Clarós-Pujol A, Pujol C, Clarós A, Wiatrow A. Long-term outcomes in Down syndrome children after cochlear implantation: particular issues and considerations. Otol Neurotol. 2019 Dec;40(10):1278–86. doi: 10.1097/MAO.0000000000002410.

Danyluk A, Jacob R. Hearing loss diagnosis and management in adults with intellectual and developmental disabilities. Adv Med. 2023 May 21;2023:6825476. doi: 10.1155/2023/6825476.

Elisha DH, Cohen DH, Monterrubio A, Hossain R, DiStefano N, Mittal R, et al. Cochlear implantation in Down syndrome: functional outcomes, challenges, and management strategies. Audiol Res. 2026 Mar 9;16(2):44. doi: 10.3390/audiolres16020044.

Joint Committee on Infant Hearing. (2019). Year 2019 position statement: Principles and guidelines for early hearing detection and intervention programs. J Early Hear Detect Interv. 44(2):1–44. <https://doi.org/10.15142/fptk-b748>

Kunst SJ, Hol MK, Cremers CW, Mylanus EA. Bone-anchored hearing aid in patients with moderate mental retardation: impact and benefit assessment. Otol Neurotol. 2007 Sep;28(6):793–7. doi: 10.1097/MAO.0b013e31809ed93a.

Määttä T, Kaski M, Taanila A, Keinänen-Kiukaanniemi S, Iivanainen M. Sensory impairments and health concerns related to the degree of intellectual disability in people with Down syndrome. Downs Syndr Res Pract. 2006 Sep;11(2):78–83. doi: 10.3104/reports.317.

Neumann K, Thomas JP, Voelter C, Dazert S. A new adhesive bone conduction hearing system effectively treats conductive hearing loss in children. Int J Pediatr Otorhinolaryngol. 2019 Jul;122:117–25. doi: 10.1016/j.ijporl.2019.03.014.

Roizen NJ, Magyar CI, Kuschner ES, Sulkes SB, Druschel C, van Wijngaarden E, et al. A community cross-sectional survey of medical problems in 440 children with Down syndrome in New York State. J Pediatr. 2014 Apr;164(4):871–5. doi: 10.1016/j.jpeds.2013.11.032.

Sait S, Alamoudi S, Zawawi F. Management outcomes of otitis media with effusion in children with down syndrome: A systematic review. Int J Pediatr Otorhinolaryngol. 2022 May;156:111092. doi: 10.1016/j.ijporl.2022.111092.

Svensk Neuropediadrisk Förening. Downs syndrom medicinskt vårdprogram 0–18 år 2014, revised 2020. [cited 2026 April 22]. Available from: <https://snpf.barnlakarforeningen.se/wp-content/uploads/sites/4/2020/06/tjugodown.pdf>

Terband H, Coppens-Hofman MC, Reffeltrath M, Maassen BAM. Effectiveness of speech therapy in adults with intellectual disabilities. J Appl Res Intellect Disabil. 2018 Mar;31(2):236–48. doi: 10.1111/jar.12384.

For the references previously indicated as “forthcoming”—as requested by the publisher—the following paper has now been published, while the others are still forthcoming or under review. Only minor revisions are still required for these, so their acceptance can be reasonably anticipated. We have therefore now labeled two of them as “under review.”

Wiegand A, Mathmann P, Wasmuth S, Prein L, Parfitt R. Scharpenberg M, et al. Clinical diagnostics after failed hearing screening in people with intellectual disabilities frequently do not occur. J Intellect Disabil Res. 2026 Apr;70(4):384-394. doi: 10.1111/jir.70078.

We have revised the following paragraph in the Introduction, as well as several smaller sections, retaining most of the earlier references while incorporating the more recent evidence:

“Ear and hearing disorders in individuals with ID are generally well treatable, especially with early and consistent intervention. Positive outcomes in auditory and speech performance following cochlear implantation in individuals with multiple disabilities, including ID, have been reported [67–69]. A recent systematic review in children [70] demonstrated improvements in environmental sound awareness, non-verbal communication, adaptive skills, QoL, and parental satisfaction, although gains in expressive language typically progress more slowly than in children without disabilities. Another systematic review revealed that cochlear implantation can significantly improve QoL and communication in children with Down syndrome [71]. Individuals with ID also benefit from bone conduction hearing aids and implants [72,73]. Hearing improvement up to normal levels has been demonstrated following consistent medical and surgical treatment of otitis media with effusion in individuals with Down syndrome [47,74]. Hearing care remains feasible and effective even in older adults with ID; after individualized acclimatization training, most individuals over 70 years were able to use hearing aids without difficulty [36]. Speech therapy has likewise been shown to improve speech intelligibility, receptive vocabulary, communicative initiative, and self-reported confidence in individuals with ID, with and without hearing loss [75].”

**Comment 4:** While the overall aim of the study was to prepare the implementation of a universal program within Germany, the results really focused on rates of hearing differences, causes of these differences and whether follow-up treatment occurred.

The study’s results are largely descriptive and quite expansive. Some of the data on the general population could be simplified. As I think about this, I think you might present global findings, % with issues, % with persistent hearing needs and how many were previously unrecognized (rather than those factors that may be amenable to treatment).

**Reply:** Thank you for these comments. They helped us identify an imprecise formulation, which we have now corrected. The sentence, “The overall aim of ~~this study~~*HörGeist* is to prepare the implementation of a universal program of regular hearing screening, intervention, and monitoring for individuals with ID in Germany,” refers to the *HörGeist* study as a whole, which is too large and complex to be fully presented in a single publication.

Therefore, the results of the study have been structured into two main papers (in addition to several secondary publications). One focuses on the prevalence of hearing loss in our cohort, stratified by age, screening setting, and type and degree of hearing loss (Mathmann et al., forthcoming)—that summarizes the requests mentioned by the reviewer. It also addresses whether such hearing loss can be detected through an outreach screening approach and evaluates the validity of this screening (e.g., sensitivity, specificity, and other performance metrics).

The second main paper (the present manuscript) addresses the central question of the overall study: to what extent the described program improved hearing care for individuals with ID compared with their status prior to participation. To this end, we assessed and compared hearing status and level of care at baseline and at study completion, detailed the types of recommended interventions and their implementation, and examined reasons for non-implementation, along with potential strategies to overcome these barriers.

Given the scope and complexity of these research questions, it was not feasible to present all aspects within a single manuscript. As is common for large and multifaceted cohort studies, the findings have therefore been distributed across several publications. We kindly ask for the reviewer’s understanding in this regard.

We would like to respectfully point out that the information requested by the reviewer was already included in the manuscript. For example, the Abstract (somewhat updated now) states:

“Hearing loss was already known in 14% of outreach participants at study entry; 8.7% had previously received hearing aids, but fewer than half (4.3%) used them. The program diagnosed hearing loss in 44% of participants, with 70% previously undiagnosed. After initial screening, 27% received therapy recommendations, but only 37% adhered to them.”

And in the Result section it is written: „Hearing loss was diagnosed in 463 of 1,053 participants (44.0% 12-month prevalence) in the outreach cohort. At baseline (T0), former or current hearing loss had been reported in 147 individuals (14.0%). Of these, the diagnosis was confirmed in 120 cases, ruled out in 24, and remained inconclusive in 3 [35]. Overall, 323 cases (69.8% of all participants with a confirmed hearing loss) represented newly detected, previously undiagnosed hearing loss… [35].”

**Comment 5:** I was hoping for a few things. One, a better understanding of the settings from which individuals were recruited and two, more evaluation of age and severity of IDD within the outcomes of hearing thresholds and treatment.

**Reply:** We thank the reviewer for these comments. The settings from which participants were recruited are presented in Table 1 with detailed numbers by age group and facility; in addition, the recruitment strategy is described in detail in the Methods section. For further details, we had also referred to our published protocol (Schwarze et al. 2023), which, however, includes only the planning aspects. We have now added Figure 2 (see below), showing the recruitment outcomes and the reasons for non-participation of facilities. We hope that all the information requested by the reviewer is now included in the manuscript.

With respect to the relationships between age and hearing loss severity, and between intellectual disability severity and hearing loss severity, as measured by hearing thresholds, we conducted a reanalysis of our data, yielding the following results which we included in the Results section:

“Hearing loss related to age, sex, and the severity of intellectual disability

An exploratory logistic regression analysis (without correction for multiplicity) examining the association of hearing loss with age, sex, and severity of ID (categorized as mild, moderate, or severe) showed that age was associated with hearing loss occurrence (adjusted odds ratio [aOR] 1.04 per year, p < .001). Higher severity of ID was also associated with an increased risk of hearing loss (moderate vs mild ID: aOR 1.71, p = .004; severe vs mild ID: aOR 2.67, p < .001), consistent with previous studies [89].”

**Comment 6:** My assumption would be that younger children may have caregivers who are more likely to address hearing status as they are still young and families are seeking improved long-term outcomes. As children age into adulthood, families may become accepting of their loved ones abilities and be okay with the “status quo”. Another example of my bias would be that I would imagine school-aged individuals in mainstream setting might be more likely to follow-through with hearing needs. Though this only included 4 individuals. There is a somewhat higher number of young children in day care settings with integration so perhaps this would be a group to compare to those in special nurseries related to treatment outcomes.

**Reply:** This assumption cannot be robustly addressed with the available data and was therefore not incorporated into the manuscript. Our observations are, in fact, mixed. Parents of young children are often overwhelmed by the high number of medical appointments and diagnostic procedures, which may lead to the neglect of less visible conditions. The reviewer’s second assumption—that children in mainstream settings receive better care than those in specialized facilities—is also not supported by our data on hearing aid provision, the most reliably traceable intervention. Of the 11 children from special nurseries or day care centers with integrative groups who were prescribed hearing aids, 8 attended special nurseries and 3 attended day care centers with integrative groups. Hearing aids were declined for one child in a special nursery and for all three children in day care centers, which, albeit in a small sample, does not support the reviewer’s hypothesis.

**Comment 7:** Page 14, line 299 has “In-in” I think this might be a typo.

**Reply:** The reviewer is right. We have corrected the typo.

**Comment 8:** Regarding the recruitment section, the authors could consider a figure that includes the 810 institutions down to 158 with the reasons for non-participation described numerically (how many refused due to time, availability, staff resources, COVID-19 policy impact (as an example, some long term care facilities in the US still limited family visitation during this time-frame).

**Reply:** We have now included the desired figure (Fig 2) in the Methods section.

**Fig 2. Recruitment outcomes for the outreach cohort and reasons for facility non-participation.**

**Comment 9:** As all of the control cohort declined participation, I might consider removing this. There were only 12 individuals presenting at clinics, I might consider removing this narrative all-together. The attrition from T0 to T1 was pretty good.

**Reply:** We kindly ask the reviewer for acknowledgement that we cannot simply disregard the intended control group. Doing so would, on the one hand, distort the study design, and on the other hand, we invested considerable effort in recruiting the control group in accordance with the study protocol (random selection by the health insurance provider, information and invitation letters, two written invitations issued by the insurers, documentation of individuals who actually presented, and efforts to obtain informed consent for participation).

However, we have moved the brief passage on non-participation to the Methods section to make it less prominent than before at the beginning of the Results section.

**Comment 10:** In the figures describing the participants, I would find Figure 2 b to be more helpful to have the groups described in comparison to themselves. For example, among those under 5, put the % of placements within that group, similarly put the school-aged together and describe % of placements within that group and so forth. I’m not thinking you need Figure 2A as you have this data in a table. I found it most helpful for the older individuals based on the relatively flat distribution across ages.

**Reply:** We prefer to keep Figure 2A because it provides the real age distribution of our sample instead of three age groups only.

As described on p. 12 of the revised manuscript (lines 275–277), our initial sample size calculation was based on age groups: group C (young children, 0–5 years), group S (school-aged children and adolescents, 6–17 years), and group A (adults, ≥18 years). We aimed to recruit approximately 350 participants per age group (see Schwarze et al. 2023).

However, because recruitment was necessarily conducted through institutions serving individuals with ID, we observed a mismatch between age and institutional setting. Many (52) children older than 5 years were still enrolled in daycare or special nursery settings, and some (34) individuals older than 18 years remained in school environments (see Table 1). As a result, age groups and their representation within institutions do not align perfectly, making it more appropriate to present these dimensions separately.

To address this, we revised Figure 2B by stratifying the bar chart by age group, such that the distribution across facilities is now presented as percentages within each age group, while also allowing identification of which facilities are predominantly associated with specific age groups. We have also revised the figure legends accordingly. We hope the reviewer will consider this a reasonable solution.

Figure 2 B now appears as follows:

**Comment 11:** On Page 17, line 379, I think it is more striking to indicate that almost half of the participants who had been prescribed amplification prior to study enrolment were not using them. This provides context for your results among those with newly identified atypical hearing thresholds. For Table 2, in the section of hearing aid devices used, it would be more helpful describe the no answer data among those who had been prescribed amplification across the groups.

**Reply:** Regarding the sentence on page 17, we have revised it to read: “…and 8.3% had previously received hearing aids, but among these, fewer than half (49.4%) were reported to use them (Table 2).”

Regarding Table 2, we thank the reviewer for this helpful comment. We have revised the data presentation to improve clarity and consistency for the reader. Specifically, all percentages related to the use of hearing aids are now calculated with respect to participants who had been prescribed hearing aids, rather than the overall study population.

**Comment 12:** The narrative in the discussion was also appropriately detailed, however I also wondered if a figure or graphic representation might display the data in a way that is easier to process for the treatment outcomes (T1). Table 3 attempts to do this. I was curious about age-based differences which would be an additional analysis. I also wondered about outcomes among those with atypical thresholds rather than just the full cohort. Both concepts are important.

**Reply:** We have now added the following new figure (Figure 4), which includes all requested information:

**Fig 5. Implementation status of prescribed hearing aids, other prescribed interventions, and the evaluation of cochlear implant candidacy at T1, stratified by age group.**

**Comment 13**: When I try to delve deeper into the data, I think there are some key findings. Among those with known atypical hearing thresholds who were using amplification, over 50% were not appropriately fit. This is a care gap.

This also builds a total % of this population with hearing needs, whether persistent or fluctuating. In the results on T1 (page 22) the authors describe hearing aids prescribed by 153. It is helpful to know if this includes those with previously known amplification needs.

See attachment for possible way to convey outcome data in a table.

**Reply:** We thank the reviewer for her careful and in-depth engagement with our data and would like to explicitly acknowledge the great effort invested in preparing this table as an exceptional contribution to the review process—one that I have rarely encountered in my own many years as Editor-in-Chief of an Elsevier journal. Thank you. Nevertheless, we are very sorry and kindly ask for your understanding that we would prefer not to adopt and include this proposed table in our manuscript. The rationale behind our consideration of this aspect is as follows:

As described in the manuscript, we were only able to ask family members or caregivers about previously or currently known hearing loss among participants in our cohort study. Considering that interviews could be conducted for only 1003 of 1053 individuals (Table 1), and that respondents often included workshop supervisors or rotating staff in daycare centers and schools, it becomes evident that the information available to respondents was frequently incomplete and not based on objective testing. Consequently, the “known hearing needs” derived from these interviews cannot be regarded as definitive; indeed, in 24 participants, hearing loss could no longer be confirmed at T0. This is reflected in the manuscript as follows: “At baseline (T0), former or current hearing loss had been reported in 147 individuals (14.0%). Of these, the diagnosis was confirmed in 120 cases, ruled out in 24, and remained inconclusive in 3 [35]. Overall, 323 cases (69.8% of all participants with a confirmed hearing loss) represented newly detected, previously undiagnosed hearing loss (missing questionnaire data: n = 20) [35].”

For this reason, when determining which individuals truly had hearing loss, we rely primarily on the meticulously collected baseline (T0) assessments rather than on interview data. It is, however, certain that 43 participants presented at T0 with hearing aids. All of this data is reported in detail in Tables 2 and 3.

The proposed table also includes therapeutic measures that are difficult to trace reliably, such as recommendations for cerumen removal. Because consultations with ENT specialists or phoniatric/pediatric audiology services often had multiple indications—for example, further diagnostic clarification when hearing assessment at T0 was incomplete—caregivers were frequently unable to report whether cerumen removal had been performed. As a result, complete data and a reliable loss-to-follow-up rate cannot be established for these measures. A similar limitation applies to the assessment of indications for ear surgery. We have described these aspects in greater detail in the Results section than would be possible in the proposed table: “No ratio between ear operations recommended and performed can be reported because indications for ear surgery were made by external ENT specialists. Furthermore, extraction of occluding cerumen by external clinical services was less well traceable due to its fluctuating status, missing documentation, or poor recall by caregivers, but this condition is of minor importance compared to the aforementioned hearing care necessities.”

We kindly ask for the reviewer’s understanding of the following: we have spent several months carefully analyzing the data and presenting them in a way that, in our view, best addresses the study’s objectives. The reviewer’s suggestion would require an entirely new data analysis, for example, stratifying therapeutic indications and implementation by age group. We have followed the majority of the reviewer’s recommendations, including conducting additional data analyses and adding four new figures. Changes between T0 and T1 are clearly presented in Tables 3 and 4. We believe that these tables continue to provide the most precise information, particularly when considered together with the newly added Figure 4 (which, in our view, captures the main information requested by the reviewer in her proposed table) and the explanations provided in the Results section. The inclusion of an additional table as proposed would not alter the main message of the paper (see response to Comment 4), but, due to the alternative mode of presentation, might disrupt the flow of arguments and interpretation for the reader.

We therefore respectfully ask the reviewer to allow us to refrain from including this table. Should the reviewer consider it essential, we would of course be willing to incorporate it; however, this would require a new data analysis (with no more clarification of mismatches between recommendations and implementation of measures) and several weeks of additional work.

**Comment 14:** I appreciate the discussion on the two individuals recommended for cochlear implantation. It would be interesting to know the rationale for declining a CI from the parent and the surgeon (anatomy, age of child, ableism, etc)

**Reply:** The precise reasons given by the physician to refuse a CI for one of the participants are not known. For the other participant, the patient experienced very high levels of anxiety; following the second assessment, it took approximately one week for her to return to her usual behavior, despite appearing cooperative and understanding during the assessment with a very empathetic audiologist. We have included this information in the revised manuscript.

**Comment 15:** Regarding Table 4, it was not clear to me how the categories were defined (high quality, un-or under treated hearing loss). I would also consider a different denominator if this table is describing those with identified hearing difference. Page 24 indicates 6 individuals were receiving high quality treatment. But not all had hearing differences, so that seems like you would want to know that by those already recognized (the n of 87). You cant access quality healthcare when you don’t know there is a hearing problem and many in your cohort had normal hearing thresholds and thus didn’t need that service (unless you are meaning regularly scheduled hearing testing).

**Reply:** Untreated hearing loss was defined as hearing loss identified at T0, T1, or in the interval between assessments, but not treated at either T0 or T1. Undertreated hearing loss referred to cases in which treatment had been initiated (e.g., hearing aids) but was insufficient, for example because devices were not worn for a sufficient number of hours per day to allow adequate amplification and habituation. In contrast, high-quality care was defined by whether hearing aid provision effectively brought heard speech into an adequately audible range and whether devices were worn for a sufficient duration each day. At T0, only six individuals met this criterion despite 43 using hearing aids. This situation improved only modestly at T1, where 12 individuals demonstrated high-quality care. These findings indicate that merely providing hearing aids to individuals with ID is insufficient; even consistent use alone is not enough. To achieve meaningful benefit, devices must be appropriately fitted to match the individual’s hearing loss—this is what we refer to as high-quality care.

Importantly, the table does not refer only to individuals with hearing loss but to the entire outreach cohort. This remains appropriate, as it allows the hearing status of each participant at T0 and T1, as well as changes between these time points, to be represented comprehensively.

Restricting the table to the 143 (not 87) individuals with previously known or suspected hearing loss out of the 1003 for whom such information was available from the interviews with caregivers) at study entry would not be meaningful. Such an approach would negate the purpose of the screening and diagnostic program, through which hearing loss was ultimately identified in 463 individuals rather than 143. We do not see a feasible way to simplify the table without compromising its informational value.

Similarly, it would not be appropriate to use as the denominator only those individuals with hearing loss at T0 or T1. Hearing status could have changed between time points—for example, transient conductive hearing loss due to otitis media with effusion may have resolved—and there were differing proportions of inconclusive hearing status at both assessments. For these reasons, a cross-tabulation based on the entire outreach cohort provides the most valid and informative representation. This approach was developed by the biomathematicians and statisticians of our project team and is methodologically well justified.

We have now modified our text as follows: “Untreated hearing loss was defined as hearing loss identified at T0, T1, or in the interval between assessments, but not treated at either T0 or T1. Undertreated hearing loss referred to cases in which treatment had been initiated (e.g., hearing aids) but was insufficient, for example because devices were not worn for a sufficient number of hours per day to allow adequate amplification and habituation. In contrast, high-quality care was defined, for example, by whether hearing aid provision effectively brought speech into an adequately audible range and whether devices were worn for a sufficient duration each day. At T0, only six individuals (0.6% of the outreach cohort) met this criterion (Table 4). This situation improved only modestly at T1, where 12 individuals (1.1%) demonstrated high-quality care, indicating persistently high rates of untreated or undertreated hearing loss despite participation in the program.”

Formularbeginn

**Comment 16:** In Table 5, I think it might be more helpful to analyze characteristics that contribute to good amplification wear-time. Additionally, Figure 4, I would be more interested in the age distribution across the hours per day of wear time. Setting may also play a role in wear-time. You could add bars within the wear-time by group (C, S, A).
On figure 4, I was also curious how the hours of amplification looked by age category. So as you have the number of participants with various wear times, can you build these into 3 bar graphs to show differences across age categories.

**Reply:** With regard to Table 5, we were unable to incorporate the information requested by the reviewer into the table. Instead, we added the following passage to the corresponding text: “According to reports from parents, relatives, and caregivers, as documented by the program-affiliated hearing-aid specialist, factors contributing to hearing-aid use included (a) close supervision by parents, educators, and teachers to ensure consistent device use (with approximately similar wearing times at home and in institutional settings for groups C and S, a tendency toward longer use at home in group C, and slightly longer use in school settings in group S), (b) the need to wear hearing aids to follow classroom instruction (group S), (c) the severity of hearing loss—greater severity being associated with a higher likelihood of use (all groups, particularly group A; the longer wearing time in private settings than in institutions in group A underlines that the most and most severe hearing loss among adults occurs in residential care settings, where most are no longer in the workforce [86])—and (d) knowledge of hearing aids among participants, their relatives, and caregivers, as well as their confidence and proficiency in handling them [96].”

Regarding Figure 6 (former figure 4), we have revised the figure to incorporate the requested information by adding a stratification by age group (C, S, A). The distribution of wear time is now displayed separately for each group.

**Comment 17:** In the discussion I appreciate the bullet points. The last bullet point could include motor abilities and cognitive abilities. It would also seem that training of staff and parents would also be an important consideration, particularly due to the low adoption of follow-up care guidance.

**Reply:** We have complemented the last bullet point as follows: "•	Insertion and removal of hearing aids, as well as battery replacement, should match the person’s individual motor and cognitive abilities."

Furthermore, we have made an adding to a bullet point before: "•	Hearing aid fitting within the everyday environment also allows a greater number of caregivers or family members to be involved in the fitting process and to be informed about the importance of consistent hearing-aid use (including agreed-upon wearing time and listening goals) and proper device cleaning; these individuals also need to be trained in the handling and use of hearing aids."

**Comment 18:** I think this work is extremely important. However the way the data is presented could be improved and highlight disparities in care for this population.

**Reply:** We thank the reviewer for highlighting the importance of this work. We have made every effort to improve the presentation of the data according to the reviewer’s suggestions; many tables and part of the figure set have been revised, two new figures have been added, and substantial new information has been incorporated into the text.

**Reviewer #2:**

**Comment 1:**

Thank you for the opportunity to review your important, well written manuscript "Overlooked and undertreated: hearing loss among individuals with intellectual disabilities – a German cohort study." This important comprehensive study is the first of its kind to implement and evaluate the feasibility of a large-scale population based program of regular hearing assessment, intervention and monitoring in community settings for individuals with intellectual disability (ID). The results document a much higher than expected rate of previously unidentified hearing loss in this population, lower rates of adoption of hearing related intervention in this population (likely due to a need to adapt health care to the unique needs of individuals with ID), and the value of community based screening compared to clinic based services. Each of these findings have important public health implications that are not well understood because of the limited prior research in this area. Thus, this study makes a significant contribution to the existing literature.

**Reply:** We are extremely grateful to the reviewer for this assessment. We feel fully understood and could not have wished for a better summary of our work. In our preliminary work, we increasingly recognized the importance and necessity of bringing the issue of under-addressed hearing loss in people with intellectual disabilities to the attention of physicians, caregivers, family members, health policymakers, economists, and society as a whole. We have learned a great deal ourselves in the process and were surprised by the high prevalence of hearing loss in this population and by the findings of our cohort study.

**Comment 2:** The manuscript could be improved by carefully reviewing to avoid implicit bias in terms utilized (e.g., please avoid terms such as "affected by intellectual disability" and "hearing impairment" which are frowned upon by these communities- instead consider "individuals with intellectual disability" and "reduced hearing" "hearing loss" or "hearing differences"). Also, please consider updating references regarding outcomes of CIs in children with DD (study cited is from 2000 and there are several updated outcome studies). Of minor note, there is a mistyped/incomplete statement line 299.

**Reply:** We thank the reviewer for these valuable comments. We have revised the manuscript to remove any potentially ableist language; although we had believed this was already the case, we did identify some somewhat insensitive formulations. As a side note, the World Health Organization, which is highly attentive to sensitive and appropriate terminology, uses the terms “hearing loss” and “hearing impairment.” In our manuscript, we have now used only the term “hearing loss.”

The reviewer is also correct that the cited literature on outcomes of cochlear implantation in children with intellectual disability required updating (as also noted by Reviewer 1). We have now revised this section and added several more recent references, as detailed in our reply to Comment 3 of Reviewer 1.

The revised passage now reads as follows: “Ear and hearing disorders in individuals with ID are generally well treatable, especially with early and consistent intervention. Positive outcomes in auditory and speech performance following cochlear implantation in individuals with multiple disabilities, including ID, have been reported [67–69]. A recent systematic review in children [70] demonstrated improvements in environmental sound awareness, non-verbal communication, adaptive skills, quality of life, and parental satisfaction, although gains in expressive language typically progress more slowly than in children without disabilities. Another systematic review revealed that cochlear implantation can significantly improve quality of life and communication in children with Down syndrome [71]. Individuals with ID also benefit from bone conduction hearing aids and implants [72,73]. Hearing improvement up to normal levels has been demonstrated following consistent medical and surgical treatment of otitis media with effusion in individuals with Down syndrome [47,74]. Hearing care remains feasible and effective even in older adults with ID; after individualized acclimatization training, most individuals over 70 years were able to use hearing aids without difficulty [36]. Speech therapy has likewise been shown to improve speech intelligibility, receptive vocabulary, communicative initiative, and self-reported confidence in individuals with ID, with and without hearing loss [75].”

As to line 299: Thank you, we have corrected the typo.

**Comment 3:** The sample and procedures are well described in the manuscript. However, please note places where the text differs from the Tables. For example, Line379 (& elsewhere in text)- reports 8.7% previously reported hearing devices prescribed which is misaligned with table 2 which reports 8.3%: similarly, ear surgery described in text as 13.9% in the table as 13.2%--- please confirm congruency of text and tables throughout.

**Reply:** We also thank the reviewer for this helpful comment. The apparent mismatch occurred only between Table 2 and the corresponding text. However, this does not reflect an error. The percentages reported in the text (8.7% and 13.9%) refer to the 1,003 individuals for whom questionnaire responses were available for these items. In contrast, Table 2 presents figures based on the entire outreach cohort (N = 1,053), counting those without questionnaire data as “no answer”. To improve consistency and clarity for readers, we have aligned the text and now refer all percentages to the full outreach cohort.

The passage reads now as follows: “At study entry (T0), caregivers reported previously or currently known hearing problems in 14.0% of the 1,053 outreach participants. A history of ear surgery was reported in 13.2% participants, and 8.3% had previously received hearing aids, but fewer than half (4.1%) were reported to use them (Table 2).”

**Comment 4:** The results and analyses are appropriately designed and interpreted and conclusions drawn from the research are appropriate and do not extend beyond the data. It was somewhat unclear in the manuscript why hearing status could not be determined in 3.3% of the sample. If logistical barriers were entirely responsible for this data that has different implications than if there are barriers in participation in traditional behavioral audiometry for individuals with ID due to a mismatch between their developmental needs and implicit task demands of audiology tests, as previously documented elsewhere in the literature. This has important potential implications and it would be helpful to further clarify.

**Reply:** This is indeed an important point, as it relates to the feasibility of the proposed program and, in a sense, to its program sensitivity and specificity, particularly if the 3.3% of individuals are considered potential carriers of hearing loss (worst-case scenario). Frankly, we were pleased and positively surprised that the proportion of individuals for whom hearing status could not be determined was so low; a priori, we had expected it to be higher. Examiner experience likely also played a role. During the first screening, 9.3% yielded no usable result, whereas this proportion had already decreased to 4.2% at the second screening one year later. The 3.3% reflects the combined assessment across both screenings, further underscoring the value of regularly repeated evaluations. The paragraph below summarizes the reasons for screenings that could not be performed or yielded non-evaluable results.

“Hearing status—and thus potential therapeutic needs—could not be reliably determined in only 35 participants (3.3% of the total sample) [35]. The reasons included refusal of testing and of manipulation of the head, restlessness, wandering around, and loud vocalizations.”

**Comment 5:** The finding that of the few participants (12) who were referred for clinical care, none consented to participate in the study could be further explored. For example, was consent sought using the same clinical staff and procedures as in community based care?

**Reply:** The reasons why the clinical screening program was taken up by so few individuals can only be inferred, but likely relate to the recruitment pathway and to the barriers that generally limit access to clinical services for individuals with intellectual disability. We have now added the following paragraphs to the Discussion section:

“Hearing screening in a clinical setting

In the control group, potential participants received invitation letters from their health insurance providers—via their families—inviting them to attend a hearing screening in a clinical setting. This recruitment pathway differed substantially from that of the outreach cohort, where institutional leaders were first contacted in writing and subsequently engaged through multiple telephone calls to emphasize the importance of the program, which they then communicated to potential participants or their caregivers. Personal interaction and sustained engagement by recruiting staff therefore likely played a key role in the success of outreach recruitment.

The reasons why only 12 control individuals were presented at clinical facilities—and none consented to study participation—can only be inferred. They most likely reflect well-documented barriers that limit access to healthcare for individuals with intellectual disability. These include the need for a second person (e.g., a caregiver) to schedule and attend appointments, often constrained by limited time and resources; the fact that hearing loss is frequently neither reported by affected individuals nor recognized by caregivers; and the tendency to assign lower priority to hearing health relative to other medical concerns or to assume that ear and hearing care has already been adequately addressed.”

**Comment 6:** Your results indicate that "Screening validity was high, with specificity and sensitivity values comparable to newborn hearing screening" but I cannot find this data reported explicitly in the text (though it could be calculated). It would be helpful to include a table with this data.

**Reply:** The HörGeist study is too large and complex to be fully presented in a single publication. Therefore, two primary papers (in addition to several secondary publications) have been prepared. One of these (Mathmann et al., forthcoming [86]) addresses the research questions related to the prevalence of hearing loss (by age, type of facility, and kind and degree of hearing loss), the ability of the outreach screening to detect hearing loss, and the validity of the screening program, particularly sensitivity and specificity. The other main paper is the manuscript presented here. We therefore kindly ask the reviewer to understand that we prefer not to include the suggested table here and instead refer to the complementary screening paper. We have, however, complemented the sentence in question in the Discussion as follows: “Screening validity was high, with a specificity of 96.4% (95% confidence interval [CI] 94.3–97.7) and a sensitivity of 98.0% (95% CI 96.1–99.0) [86], values comparable to those of newborn hearing screening [90]”.

[90] refers to Butcher E, Dezateux C, Cortina-Borja M, Knowles RL. Prevalence of permanent childhood hearing loss detected at the universal newborn hearing screen: systematic review and meta-analysis. PLoS One. 2019 Jul;14(7):e0219600. doi: 10.1371/journal.pone.0219600.

**Comment 7:** I particularly appreciated attention paid in the discussion to attempt to understand reasons for reduced follow up/adherence to recommended audiological intervention, including technology use, and efforts to explain unique developmental considerations in supporting individuals with ID with hearing technology use. The authors appropriately addressed these considerations without blaming caregivers or participants. The findings highlight the likelihood that misunderstanding the developmental needs of individuals with ID may contribute to lower than expected adoption rates of technology, which is important context as the data as written could be misinterpreted as suggesting that there is no utility in screening when it does not ultimately result in increased adoption of or access to high quality health care.

**Reply:** We are most grateful for this comment. It was challenging to strike the right balance in presenting the current situation clearly, weighing the barriers, and offering a hopeful outlook, while maintaining sensitivity and understanding for individuals with ID, their caregivers, and all others involved.

**Comment 8:** Finally, I think the manuscript could be improved with some discussion regarding implications of these findings within a a global context. For example, this study has important implications in countries where universal health care is not readily available and where hearing technology may not be covered by public insurance particularly for adults. Therefore, given the high prevalence of unidentified hearing loss in this sample, screening prior to transitioning to adult based health care becomes even more important for individuals with ID. Additionally, there are currently no protocols for screening hearing in the ID population specifically beyond the newborn period, despite evidence of this strong co-occurrence. Some discussion about these implications would improve the discussion.

**Reply:** We thank the reviewer for this important comment. Indeed, our study addresses a key public health issue that we hope will be of broad international interest. We have therefore added the following passage to the Discussion:

“At an international level, our findings suggest that repeated screening and intervention programs generate learning effects among screening personnel, resulting in fewer inconclusive findings (in our study 13% at T0 vs. 6.6% at T1), reduced referrals for external diagnostics (in our study 30% vs. 24%), and improved screening performance and diagnostic confidence. Screening intervals should be short in early childhood, when timely detection and intervention are most critical, particularly in low-resource settings where child health is often prioritized over adults. Intervals should also reflect the age-related increase in hearing loss prevalence, especially among individuals with Down syndrome. We therefore recommend an approach aligned with the EFAS Audiology & Intellectual Disability Workgroup [77], including newborn hearing screening, close follow-up intervals up to six years of age, and more frequent monitoring for individuals with Down syndrome (Table 6). Countries should prioritize the implementation of such programs, alongside accessible rehabilitation strategies that emphasize communication and hearing training in partnership with caregivers.”

**Table 6. Recommendations for audiological screening and management for individuals with intellectual disability, modified from [77].**

| **Individuals with ID in general** | **Individuals with Down syndrome** | **Individuals with ID eligible for hearing devices** |
| --- | --- | --- |
| Neonatal hearing screening | | Annual hearing evaluation |
| Annual screening < age 6 | 2x/year > age 6 |  |
| Every 3 years from age 6 to 18 | Every 2 years from age 6 to 18 |  |
| Every 5 years from age 18 to 50 | Every 3 years from age 18 and 35 |  |
| Every 3 years > age 50 | Annual > age 35 |  |
| Annually if 8h/day or more noise exposure (>80 dBA) | | |

**Comment 9:**

Though not a critique of the current study, future studies would benefit from inclusion of quality of life measures and participation ratings of participants/their caregivers comparing individuals with ID with vs without hearing differences.

**Reply:** As noted in our response to Comment 2 of Reviewer 1, we were fortunately able within the project to develop a questionnaire instrument to assess hearing-related quality of life in individuals with intellectual disability based on caregiver reports. This QoL score decreased significantly with increasing severity of hearing loss according to caregiver ratings—even when caregivers were unaware of the degree of hearing loss (Zielonkowski et al. 2025 [35]).

We have now inserted the following passage in the Introduction section:

“Owing to the heterogeneity in phenotype, severity, and overall impact of ID, the additional effect of co-occurring hearing loss is difficult to quantify. While in typically developing children, established developmental trajectories in auditory, language, and social domains allow the impact of hearing loss to be benchmarked against normative data, in individuals with ID, by contrast, the relative contribution of ID and hearing loss to language and behavioral development, independence, and social participation varies widely, and even the onset of hearing loss is often unknown. Yet, in a separate part of our study, we developed a caregiver-reported questionnaire to assess hearing-related quality of life (QoL) in individuals with ID [35]. Multivariable regression analysis showed a small but significant negative association between the degree of hearing loss and hearing-related QoL (β = −0.069; *p* < .001; adjusted R² = .081), adjusted for age, sex, and severity of ID, suggesting that hearing-related QoL may capture the impact of hearing loss and potentially also of hearing care.”

6. PLOS authors have the option to publish the peer review history of their article ([what does this mean?](https://track.editorialmanager.com/CL0/https:%2F%2Fjournals.plos.org%2Fglobalpublichealth%2Fs%2Feditorial-and-peer-review-process%23loc-peer-review-history/1/010f019d616b7b50-d99fae09-5dca-4ab6-ae70-24180f897a79-000000/5OipEKsRA9LpSWcIJRyRgYtX4A8Qnae1DVAyxbdWHVY=257)). If published, this will include your full peer review and any attached files.


**Do you want your identity to be public for this peer review?** If you choose “no”, your identity will remain anonymous but your review may still be made public.


For information about this choice, including consent withdrawal, please see our [Privacy Policy](https://track.editorialmanager.com/CL0/https:%2F%2Fwww.plos.org%2Fprivacy-policy/1/010f019d616b7b50-d99fae09-5dca-4ab6-ae70-24180f897a79-000000/1xB6lOp_lEUah3pz-3-6-kuK4iu1xVO9PlTIXdc3awc=257).

Reviewer #1: **Yes:** Susan Wiley

Reviewer #2: No

 [NOTE: If reviewer comments were submitted as an attachment file, they will be attached to this email and accessible via the submission site. Please log into your account, locate the manuscript record, and check for the action link "View Attachments". If this link does not appear, there are no attachment files.]

Figure Resubmissions:

While revising your submission, we strongly recommend that you use PLOS’s NAAS tool (<https://ngplosjournals.pagemajik.ai/artanalysis>) to test your figure files. NAAS can convert your figure files to the TIFF file type and meet basic requirements (such as print size, resolution), or provide you with a report on issues that do not meet our requirements and that NAAS cannot fix.

After uploading your figures to PLOS’s NAAS tool - <https://ngplosjournals.pagemajik.ai/artanalysis>, NAAS will process the files provided and display the results in the "Uploaded Files" section of the page as the processing is complete. If the uploaded figures meet our requirements (or NAAS is able to fix the files to meet our requirements), the figure will be marked as "fixed" above. If NAAS is unable to fix the files, a red "failed" label will appear above. When NAAS has confirmed that the figure files meet our requirements, please download the file via the download option, and include these NAAS processed figure files when submitting your revised manuscript.

*In compliance with data protection regulations, you may request that we remove your personal registration details at any time.* [*(Remove my information/details)*](https://track.editorialmanager.com/CL0/https:%2F%2Fwww.editorialmanager.com%2Fpgph%2Flogin.asp%3Fa=r/1/010f019d616b7b50-d99fae09-5dca-4ab6-ae70-24180f897a79-000000/bcFJXsvoiyqJAqjn79RPVLV6DZgRL4_3VKo5fd_7dQc=257)*. Please contact the publication office if you have any questions.*
