# Supplementary material for: Overlooked and undertreated: Hearing loss among individuals with intellectual disabilities – A German cohort study
Source: PLOS Glob Public Health. 2026 Aug 19;6(8):e0006872. doi: 10.1371/journal.pgph.0006872 (PMC13489453; doi:10.1371/journal.pgph.0006872)
Supplement: Peer review history

# Author comment: Author response to Decision Letter 0

**DOI:** 10.1371/journal.pgph.0006872.r001

11 Feb 2026

Attachment Submitted filename: Rebuttal letter.pdf

# Peer review: Decision Letter 0

**DOI:** 10.1371/journal.pgph.0006872.r002

**Authors:** Jen Edwards

6 Apr 2026

PGPH-D-26-00483

Overlooked and undertreated: hearing loss among individuals with intellectual disabilities – a German cohort study

PLOS Global Public Health

Dear Dr. Neumann,

Thank you for submitting your manuscript to PLOS Global Public Health. After careful consideration, we feel that it has merit but does not fully meet PLOS Global Public Health’s publication criteria as it currently stands. Therefore, we invite you to submit a revised version of the manuscript that addresses the points raised during the review process.

The manuscript has been evaluated by two reviewers, and their comments are available below. The reviewers have raised a number of major concerns. They request some language revision to avoid implicit bias, as well as updates to the literature review. They also request clarification in a number of areas and updates to ensure the reporting of results in the text is in line with that presented in figures and tables. Could you please carefully revise the manuscript to address all comments raised?

Please submit your revised manuscript by May 17 2026 11:59PM. If you will need more time than this to complete your revisions, please reply to this message or contact the journal office at globalpubhealth@plos.org. When you're ready to submit your revision, log on to https://www.editorialmanager.com/pgph/ and select the 'Submissions Needing Revision' folder to locate your manuscript file.

Please include the following items when submitting your revised manuscript:

- A letter that responds to each point raised by the editor and reviewer(s). You should upload this letter as a separate file labeled 'Response to Reviewers'.
- A marked-up copy of your manuscript that highlights changes made to the original version. You should upload this as a separate file labeled 'Revised Manuscript with Track Changes'.
- An unmarked version of your revised paper without tracked changes. You should upload this as a separate file labeled 'Manuscript'.

Guidelines for resubmitting your figure files are available below the reviewer comments at the end of this letter.

As the corresponding author, your ORCID iD is verified in the submission system and will appear in the published article. PLOS supports the use of ORCID, and we encourage all coauthors to register for an ORCID iD and use it as well. Please encourage your coauthors to verify their ORCID iD within the submission system before final acceptance, as unverified ORCID iDs will not appear in the published article. *Only*  the individual author can complete the verification step; PLOS staff *cannot*  verify ORCID iDs on behalf of authors.

We look forward to receiving your revised manuscript.

Kind regards,

Jen Edwards

Staff Editor

PLOS Global Public Health

Journal Requirements:

1. Please provide a detailed online Financial Disclosure statement. This is published with the article. It must therefore be completed in full sentences and contain the exact wording you wish to be published.

a) State the initials, alongside each funding source, of each author to receive each grant. For example: “This work was supported by the National Institutes of Health (####### to AM; ###### to CJ) and the National Science Foundation (###### to AM).”

b) State what role the funders took in the study. If the funders had no role in your study, please state: “The funders had no role in study design, data collection and analysis, decision to publish, or preparation of the manuscript.”

For more information, please go to our submission guidelines:

https://journals.plos.org/globalpublichealth/s/submission-guidelines#loc-financial-disclosure-statement

2. Please ensure that the funders and grant numbers match between the Financial Disclosure field and the Funding Information tab in your submission form. Note that the funders must be provided in the same order in both places as well.

3. When completing the data availability statement of the submission form, you indicated that you will make your data available on acceptance. We strongly recommend all authors decide on a data sharing plan before acceptance, as the process can be lengthy and hold up publication timelines. Please note that, though access restrictions are acceptable now, your entire data will need to be made freely accessible if your manuscript is accepted for publication. This policy applies to all data except where public deposition would breach compliance with the protocol approved by your research ethics board. If you are unable to adhere to our open data policy, please kindly revise your statement to explain your reasoning and we will seek the editor's input on an exemption. Please be assured that, once you have provided your new statement, the assessment of your exemption will not hold up the peer review process.

For further assistance, you may go to: http://journals.plos.org/globalpublichealth/s/data-availability

4. If the reviewer comments include a recommendation to cite specific previously published works, please review and evaluate these publications to determine whether they are relevant and should be cited. There is no requirement to cite these works unless the editor has indicated otherwise.

Additional Editor Comments (if provided):

[Note: HTML markup is below. Please do not edit.]

Reviewers' comments:

Reviewer's Responses to Questions

**Comments to the Author**

1. Does this manuscript meet PLOS Global Public Health’s publication criteria? Is the manuscript technically sound, and do the data support the conclusions? The manuscript must describe methodologically and ethically rigorous research with conclusions that are appropriately drawn based on the data presented.

Reviewer #1: Yes

Reviewer #2: Yes

**********

2. Has the statistical analysis been performed appropriately and rigorously?

Reviewer #1: N/A

Reviewer #2: Yes

**********

3. Have the authors made all data underlying the findings in their manuscript fully available (please refer to the Data Availability Statement at the start of the manuscript PDF file)?

The PLOS Data policy requires authors to make all data underlying the findings described in their manuscript fully available without restriction, with rare exception. The data should be provided as part of the manuscript or its supporting information, or deposited to a public repository. For example, in addition to summary statistics, the data points behind means, medians and variance measures should be available. If there are restrictions on publicly sharing data—e.g. participant privacy or use of data from a third party—those must be specified.

Reviewer #1: Yes

Reviewer #2: Yes

**********

4. Is the manuscript presented in an intelligible fashion and written in standard English?

PLOS Global Public Health does not copyedit accepted manuscripts, so the language in submitted articles must be clear, correct, and unambiguous. Any typographical or grammatical errors should be corrected at revision, so please note any specific errors here.

Reviewer #1: Yes

Reviewer #2: Yes

**********

5. Review Comments to the Author

Please use the space provided to explain your answers to the questions above. You may also include additional comments for the author, including concerns about dual publication, research ethics, or publication ethics. (Please upload your review as an attachment if it exceeds 20,000 characters)

Reviewer #1: see uploaded document example of table.

The paper, Overlooked and undertreated, hearing loss among individual with IDD is an important contribution to the literature. There is very little published on rates of abnormal hearing thresholds among adults with IDD.

The authors have developed an important program across Germany that provides strong guidance on the unmet health care needs of children and adults with intellectual disability.

This paper would benefit from streamlining a bit within the background section and results as it is lengthy and sometimes with quite detailed. For example in the background, causes of hearing differences among individuals with ID, could be more concise. If there is literature on the impact of hearing differences in IDD that would also provide more impetus for the reader to want to follow hearing and outer and middle ear issues. It is possible that within this journal, the background should be expansive. I am more accustomed to medical writing where the discussion is the section with most in-depth information.

While I can see considerable work and depth of their literature review, some references were quite old and there may be newer publications that could be presented instead. There were also publications that were described as “forthcoming”. I am also not familiar with this approach. If a paper is in press or epub ahead of print, that makes sense, but a publication in preparation would typically be described as a communication with the authors. One example of a citation as forthcoming was #72. I think at the time of manuscript preparation this was e pub ahead (as of January 2026) and is now published as of April 2026. Another example of an older citation is the AAP guidelines for the medical care of Down syndrome. This has been updated as of 2022 and there is now an adult healthcare guidelines for individuals with Trisomy 21. There is also now a JCIH statement as of 2019 which is not pubmed queriable (JEHDI). Some of the cochlear implant citations are old (performance of multiply handicapped children using CI’s.

While the overall aim of the study was to prepare the implementation of a universal program within Germany, the results really focused on rates of hearing differences, causes of these differences and whether follow-up treatment occurred.

The study’s results are largely descriptive and quite expansive. Some of the data on the general population could be simplified. As I think about this, I think you might present global findings, % with issues, % with persistent hearing needs and how many were previously unrecognized (rather than those factors that may be amenable to treatment).

I was hoping for a few things. One, a better understanding of the settings from which individuals were recruited and two, more evaluation of age and severity of IDD within the outcomes of hearing thresholds and treatment. My assumption would be that younger children may have caregivers who are more likely to address hearing status as they are still young and families are seeking improved long-term outcomes. As children age into adulthood, families may become accepting of their loved ones abilities and be okay with the “status quo”. Another example of my bias would be that I would imagine school-aged individuals in mainstream setting might be more likely to follow-through with hearing needs. Though this only included 4 individuals. There is a somewhat higher number of young children in day care settings with integration so perhaps this would be a group to compare to those in special nurseries related to treatment outcomes.

Page 14, line 299 has “In-in” I think this might be a typo.

Regarding the recruitment section, the authors could consider a figure that includes the 810 institutions down to 158 with the reasons for non-participation described numerically (how many refused due to time, availability, staff resources, COVID-19 policy impact (as an example, some long term care facilities in the US still limited family visitation during this time-frame).

As all of the control cohort declined participation, I might consider removing this. There were only 12 individuals presenting at clinics, I might consider removing this narrative all-together. The attrition from T0 to T1 was pretty good.

In the figures describing the participants, I would find Figure 2 b to be more helpful to have the groups described in comparison to themselves. For example, among those under 5, put the % of placements within that group, similarly put the school-aged together and describe % of placements within that group and so forth. I’m not thinking you need Figure 2A as you have this data in a table. I found it most helpful for the older individuals based on the relatively flat distribution across ages.

On Page 17, line 379, I think it is more striking to indicate that almost half of the participants who had been prescribed amplification prior to study enrolment were not using them. This provides context for your results among those with newly identified atypical hearing thresholds. For Table 2, in the section of hearing aid devices used, it would be more helpful describe the no answer data among those who had been prescribed amplification across the groups.

The narrative in the discussion was also appropriately detailed, however I also wondered if a figure or graphic representation might display the data in a way that is easier to process for the treatment outcomes (T1). Table 3 attempts to do this. I was curious about age-based differences which would be an additional analysis. I also wondered about outcomes among those with atypical thresholds rather than just the full cohort. Both concepts are important.

When I try to delve deeper into the data, I think there are some key findings. Among those with known atypical hearing thresholds who were using amplification, over 50% were not appropriately fit. This is a care gap.

This also builds a total % of this population with hearing needs, whether persistent or fluctuating. In the results on T1 (page 22) the authors describe hearing aids prescribed by 153. It is helpful to know if this includes those with previously known amplification needs.

See attachment for possible way to convey outcome data in a table.

I appreciate the discussion on the two individuals recommended for cochlear implantation. It would be interesting to know the rationale for declining a CI from the parent and the surgeon (anatomy, age of child, ableism, etc)

Regarding Table 4, it was not clear to me how the categories were defined (high quality, un-or under treated hearing loss). I would also consider a different denominator if this table is describing those with identified hearing difference. Page 24 indicates 6 individuals were receiving high quality treatment. But not all had hearing differences, so that seems like you would want to know that by those already recognized (the n of 87). You cant access quality healthcare when you don’t know there is a hearing problem and many in your cohort had normal hearing thresholds and thus didn’t need that service (unless you are meaning regularly scheduled hearing testing).

In Table 5, I think it might be more helpful to analyze characteristics that contribute to good amplification wear-time. Additionally, Figure 4, I would be more interested in the age distribution across the hours per day of wear time. Setting may also play a role in wear-time. You could add bars within the wear-time by group (C, S, A).

On figure 4, I was also curious how the hours of amplification looked by age category. So as you have the number of participants with various wear times, can you build these into 3 bar graphs to show differences across age categories.

In the discussion I appreciate the bullet points. The last bullet point could include motor abilities and cognitive abilities. It would also seem that training of staff and parents would also be an important consideration, particularly due to the low adoption of follow-up care guidance.

I think this work is extremely important. However the way the data is presented could be improved and highlight disparities in care for this population.

Reviewer #2: Thank you for the opportunity to review your important, well written manuscript "Overlooked and undertreated: hearing loss among individuals with intellectual disabilities – a German cohort study." This important comprehensive study is the first of its kind to implement and evaluate the feasibility of a large-scale population based program of regular hearing assessment, intervention and monitoring in community settings for individuals with intellectual disability (ID). The results document a much higher than expected rate of previously unidentified hearing loss in this population, lower rates of adoption of hearing related intervention in this population (likely due to a need to adapt health care to the unique needs of individuals with ID), and the value of community based screening compared to clinic based services. Each of these findings have important public health implications that are not well understood because of the limited prior research in this area. Thus, this study makes a significant contribution to the existing literature.

The manuscript could be improved by carefully reviewing to avoid implicit bias in terms utilized (e.g., please avoid terms such as "affected by intellectual disability" and "hearing impairment" which are frowned upon by these communities- instead consider "individuals with intellectual disability" and "reduced hearing" "hearing loss" or "hearing differences"). Also, please consider updating references regarding outcomes of CIs in children with DD (study cited is from 2000 and there are several updated outcome studies). Of minor note, there is a mistyped/incomplete statement line 299.

The sample and procedures are well described in the manuscript. However, please note places where the text differs from the Tables. For example, Line379 (& elsewhere in text)- reports 8.7% previously reported hearing devices prescribed which is misaligned with table 2 which reports 8.3%: similarly, ear surgery described in text as 13.9% in the table as 13.2%--- please confirm congruency of text and tables throughout.

The results and analyses are appropriately designed and interpreted and conclusions drawn from the research are appropriate and do not extend beyond the data. It was somewhat unclear in the manuscript why hearing status could not be determined in 3.3% of the sample. If logistical barriers were entirely responsible for this data that has different implications than if there are barriers in participation in traditional behavioral audiometry for individuals with ID due to a mismatch between their developmental needs and implicit task demands of audiology tests, as previously documented elsewhere in the literature. This has important potential implications and it would be helpful to further clarify.

The finding that of the few participants (12) who were referred for clinical care, none consented to participate in the study could be further explored. For example, was consent sought using the same clinical staff and procedures as in community based care?

Your results indicate that "Screening validity was high, with specificity and sensitivity values comparable to newborn

hearing screening" but I cannot find this data reported explicitly in the text (though it could be calculated). It would be helpful to include a table with this data.

I particularly appreciated attention paid in the discussion to attempt to understand reasons for reduced follow up/adherence to recommended audiological intervention, including technology use, and efforts to explain unique developmental considerations in supporting individuals with ID with hearing technology use. The authors appropriately addressed these considerations without blaming caregivers or participants. The findings highlight the likelihood that misunderstanding the developmental needs of individuals with ID may contribute to lower than expected adoption rates of technology, which is important context as the data as written could be misinterpreted as suggesting that there is no utility in screening when it does not ultimately result in increased adoption of or access to high quality health care.

Finally, I think the manuscript could be improved with some discussion regarding implications of these findings within a a global context. For example, this study has important implications in countries where universal health care is not readily available and where hearing technology may not be covered by public insurance particularly for adults. Therefore, given the high prevalence of unidentified hearing loss in this sample, screening prior to transitioning to adult based health care becomes even more important for individuals with ID. Additionally, there are currently no protocols for screening hearing in the ID population specifically beyond the newborn period, despite evidence of this strong co-occurrence. Some discussion about these implications would improve the discussion.

Though not a critique of the current study, future studies would benefit from inclusion of quality of life measures and participation ratings of participants/their caregivers comparing individuals with ID with vs without hearing differences.

**********

6. PLOS authors have the option to publish the peer review history of their article (what does this mean?). If published, this will include your full peer review and any attached files.

**Do you want your identity to be public for this peer review?**  If you choose “no”, your identity will remain anonymous but your review may still be made public.

For information about this choice, including consent withdrawal, please see our Privacy Policy.

Reviewer #1: **Yes:** Susan Wiley

Reviewer #2: No

**********

[NOTE: If reviewer comments were submitted as an attachment file, they will be attached to this email and accessible via the submission site. Please log into your account, locate the manuscript record, and check for the action link "View Attachments". If this link does not appear, there are no attachment files.]

-->Figure Resubmissions:

While revising your submission, we strongly recommend that you use PLOS’s NAAS tool (https://ngplosjournals.pagemajik.ai/artanalysis) to test your figure files. NAAS can convert your figure files to the TIFF file type and meet basic requirements (such as print size, resolution), or provide you with a report on issues that do not meet our requirements and that NAAS cannot fix.

After uploading your figures to PLOS’s NAAS tool - https://ngplosjournals.pagemajik.ai/artanalysis, NAAS will process the files provided and display the results in the "Uploaded Files" section of the page as the processing is complete. If the uploaded figures meet our requirements (or NAAS is able to fix the files to meet our requirements), the figure will be marked as "fixed" above. If NAAS is unable to fix the files, a red "failed" label will appear above. When NAAS has confirmed that the figure files meet our requirements, please download the file via the download option, and include these NAAS processed figure files when submitting your revised manuscript.

Attachment Submitted filename: PLOS Global public health review table example.docx

# Author comment: Author response to Decision Letter 1

**DOI:** 10.1371/journal.pgph.0006872.r003

5 Jun 2026

Attachment Submitted filename: Response to Reviewers.docx

# Peer review: Decision Letter 1

**DOI:** 10.1371/journal.pgph.0006872.r004

**Authors:** Julia Robinson

30 Jun 2026

Overlooked and undertreated: hearing loss among individuals with intellectual disabilities – a German cohort study

PGPH-D-26-00483R1

Dear Prof. Neumann,

We are pleased to inform you that your manuscript 'Overlooked and undertreated: hearing loss among individuals with intellectual disabilities – a German cohort study' has been provisionally accepted for publication in PLOS Global Public Health.

Before your manuscript can be formally accepted you will need to complete some formatting changes, which you will receive in a follow up email. A member of our team will be in touch with a set of requests.

Please note that your manuscript will not be scheduled for publication until you have made the required changes, so a swift response is appreciated.

IMPORTANT: The editorial review process is now complete. PLOS will only permit corrections to spelling, formatting or significant scientific errors from this point onwards. Requests for major changes, or any which affect the scientific understanding of your work, will cause delays to the publication date of your manuscript.

If your institution or institutions have a press office, please notify them about your upcoming paper to help maximize its impact. If they'll be preparing press materials, please inform our press team as soon as possible -- no later than 48 hours after receiving the formal acceptance. Your manuscript will remain under strict press embargo until 2 pm Eastern Time on the date of publication. For more information, please contact globalpubhealth@plos.org.

Thank you again for supporting Open Access publishing; we are looking forward to publishing your work in PLOS Global Public Health.

Best regards,

Julia Robinson

Executive Editor

PLOS Global Public Health

***********************************************************

Reviewer Comments (if any, and for reference):

Reviewer's Responses to Questions

**Comments to the Author**

1. If the authors have adequately addressed your comments raised in a previous round of review and you feel that this manuscript is now acceptable for publication, you may indicate that here to bypass the “Comments to the Author” section, enter your conflict of interest statement in the “Confidential to Editor” section, and submit your "Accept" recommendation.

Reviewer #1: All comments have been addressed

**********

2. Does this manuscript meet PLOS Global Public Health’s publication criteria? Is the manuscript technically sound, and do the data support the conclusions? The manuscript must describe methodologically and ethically rigorous research with conclusions that are appropriately drawn based on the data presented.

Reviewer #1: Yes

**********

3. Has the statistical analysis been performed appropriately and rigorously?

Reviewer #1: Yes

**********

4. Have the authors made all data underlying the findings in their manuscript fully available (please refer to the Data Availability Statement at the start of the manuscript PDF file)?

The PLOS Data policy requires authors to make all data underlying the findings described in their manuscript fully available without restriction, with rare exception. The data should be provided as part of the manuscript or its supporting information, or deposited to a public repository. For example, in addition to summary statistics, the data points behind means, medians and variance measures should be available. If there are restrictions on publicly sharing data—e.g. participant privacy or use of data from a third party—those must be specified.

Reviewer #1: Yes

**********

5. Is the manuscript presented in an intelligible fashion and written in standard English?

PLOS Global Public Health does not copyedit accepted manuscripts, so the language in submitted articles must be clear, correct, and unambiguous. Any typographical or grammatical errors should be corrected at revision, so please note any specific errors here.

Reviewer #1: Yes

**********

6. Review Comments to the Author

Please use the space provided to explain your answers to the questions above. You may also include additional comments for the author, including concerns about dual publication, research ethics, or publication ethics. (Please upload your review as an attachment if it exceeds 20,000 characters)

Reviewer #1: Thank you for your work.

**********

7. PLOS authors have the option to publish the peer review history of their article (what does this mean?). If published, this will include your full peer review and any attached files.

**Do you want your identity to be public for this peer review?**  If you choose “no”, your identity will remain anonymous but your review may still be made public.

For information about this choice, including consent withdrawal, please see our Privacy Policy.

Reviewer #1: **Yes:** Susan Wiley

**********
